# Enrichment and Identification of the Most Abundant Zinc Binding Proteins in Developing Barley Grains by Zinc-IMAC Capture and Nano LC-MS/MS

**DOI:** 10.3390/proteomes6010003

**Published:** 2018-01-17

**Authors:** Giuseppe Dionisio, Mohammad Nasir Uddin, Eva Vincze

**Affiliations:** Department of Molecular Biology and Genetics, Faculty of Science and Technology, Research Centre Flakkebjerg, Aarhus University, 4200 Slagelse, Denmark; nasiru32@gmail.com

**Keywords:** metal-binding proteins, Zn-IMAC, mass spectrometry

## Abstract

*Background*: Zinc accumulates in the embryo, aleurone, and subaleurone layers at different amounts in cereal grains. Our hypothesis is that zinc could be stored bound, not only to low MW metabolites/proteins, but also to high MW proteins as well. *Methods*: In order to identify the most abundant zinc binding proteins in different grain tissues, we microdissected barley grains into (1) seed coats; (2) aleurone/subaleurone; (3) embryo; and (4) endosperm. Initial screening for putative zinc binding proteins from the different tissue types was performed by fractionating proteins according to solubility (Osborne fractionation), and resolving those via Sodium Dodecyl Sulfate Polyacrylamide Gel Electrophoresis (SDS-PAGE) followed by polyvinylidene fluoride (PVDF) membrane blotting and dithizone staining. Selected protein fractions were subjected to Zn^2+^-immobilized metal ion affinity chromatography, and the captured proteins were identified using nanoscale liquid chromatography coupled to tandem mass spectrometry (nanoLC-MS/MS). *Results*: In the endosperm, the most abundant zinc binding proteins were the storage protein B-hordeins, gamma-, and D-hordeins, while in the embryo, 7S globulins storage proteins exhibited zinc binding. In the aleurone/subaleurone, zinc affinity captured proteins were late abundant embryogenesis proteins, dehydrins, many isoforms of non-specific lipid transfer proteins, and alpha amylase trypsin inhibitor. *Conclusions*: We have shown evidence that abundant barley grain proteins have been captured by Zn-IMAC, and their zinc binding properties in relationship to the possibility of zinc storage is discussed.

## 1. Introduction

Zinc is an essential micronutrient for plants and animals, and is considered the most abundant transition metal in organisms after iron [1]. In biological systems, Zn is known to be associated/bound with proteins having different structural and functional roles [2]. An in silico study revealed that, in humans, about 10% of proteins (i.e., 2800) could potentially bind Zn [1]. In *Arabidopsis*, 2367 proteins in 181 gene families were identified as having affinity for Zn [1,3]. Although a lot of zinc binding motifs are possible to be predicted by bioinformatics analyses, the total number of actual zinc binding proteins might be higher [4]. Furthermore, in addition to known zinc binding epitopes/motifs in protein sequences, there are intermolecular binding sites (in which Zn acts as a bridging ligand between two polypeptides) which are extremely difficult to predict in silico [3,5].

In the developing world, up to 75% of the daily calorie intake is derived from cereals which have very low Zn bioavailability (www.harvestplus.org). Therefore, human Zn deficiency is prevalent in areas with high cereal food consumption, and is considered as one of the top priority micronutrient deficiency problems [6,7].

Plant protein characterization is traditionally based on their extraction and solubility in water (albumins), dilute saline (globulins), alcohol/water mixtures (prolamins), and dilute acid or alkali (glutelins) [8,9]. This so-called Osborne fractionation procedure is applied to milled seed material, which is extracted sequentially with water to extract albumins, dilute saline (0.5–1.0 M NaCl) to extract globulins, alcohol–water mixtures (traditionally 60–70% (*v*/*v*) ethanol) to extract prolamins, and dilute acid, or alkali to extract glutelins [10,11]. However, nowadays, a modified version of Osborne fractionation has been used by the replacement of ethanol with other alcohols (e.g., 50% (*v*/*v*) propanol), and the addition of a reducing agent to extract prolamin subunits present in polymers associated by interchain disulfide bonds [10]. Although “Osborne fractionation” is still widely used for classification, at the present time, grain proteins are divided into three groups: storage proteins, structural and metabolic proteins, and protective proteins [12]. The largest group, the storage proteins, are present in high amounts, and play an important biological role during seed development and germination [13,14]. The major grain storage proteins include albumins, globulins, and prolamins (called hordeins in barley) [15]. In cereals, prolamins represent the major grain storage protein, except rice and oat, where the major storage proteins are globulins [11,13]. An important role of the storage proteins in zinc binding has been recently suggested by the finding of a positive linear relationship between the increase of barley major prolamin fraction, B-hordein, and total grain Zn concentration [16].

There is a close relationship between the amount of Zn in a grain and its protein content [17,18]. Zn and protein content in the embryo and aleurone layer are higher than in the endosperm [19,20]. In grains, the micronutrients have a peripheral gradient distribution. The six sub layers/fractions of the testa–aleurone–endosperm interfaces shows a decreased micronutrient concentration directed towards the endosperm [21]. During the milling process, the embryo and aleurone layers are removed, and as a consequence, a significant amount of the initial Zn content is lost from the cereal based food [22]. Furthermore, in the future, the predicted increase in CO_2_ concentration will have the effect of lowering protein concentrations and increasing the carbohydrate content of the grains. As a further consequence, the C3 grains and legumes will have lower concentrations of zinc when grown under field conditions at the elevated atmospheric CO_2_ level [23].

The supply of minerals to the developing cereal grain can come from two sources: (1) direct uptake from the soil and/or (2) the remobilization of stored minerals in leaves as they senesce during grain filling [24]. The Zn fluxes derived from uptake and remobilization are almost equal in plants with low Zn status, while at high Zn status, plant remobilization delivers four times more zinc to the developing grains than the root Zn uptake [25]. Uptake of Zn^2+^ ions from the rhizosphere is the first step for accumulating into the plant to translocation to seeds. Plant-mediated acidification and secretion of low molecular weight organic chelators may play a role in Zn solubilization in the rhizosphere. Metal-binding proteins and low molecular weight metal chelators (e.g., phytochelatins, nicotianamine, and metallothioneins) in the cytosol work either as (i) metal chaperones—which interact with and donate metals to apo-metalloproteins or transport proteins that mediate the sequestration or efflux of metal ions; or (ii) intracellular metal buffers—which keep the free metal–ion concentration very low [26]. The hypothesis that phytic acid could be the main low molecular sink ligand to bind and chelate zinc was investigated by metallomics speciation analysis, using SEC-ICP-MS and IP-ICP-MS [19], and synchrotron-based X-ray fluorescence and absorption analysis [27]. The outcome of the two studies are controversial: the first investigation excluded the suggested role of phytic acid as ligand, while the second study proposed that phytic acid plays important role as zinc binding ligand in cereals. The facts that phytic acid to Zn molar ratio decreases with zinc fertilization, while protein content increases, supports the hypothesis that some proteins are more important in Zn binding than phytic acid [28,29]. Furthermore, in the grain, phytic acid localizes in the protein storage vacuoles of the aleurone cells, and the globoids do not contain zinc, as measured by ICP-MS [30,31,32]. On the other hand, increased phytochelatin synthesis correlates with zinc accumulation in vegetative vacuoles only [33]. During grain filling, zinc should be stored in ligands with high zinc storage capability per molecule (i.e., metallothioneins). Enhanced levels of Zn can only be achieved when additional sink molecules/peptides/proteins are de novo synthesized in the grain tissue [34]. Previous studies on the movement of zinc in cereal grain focused on the Zn transporter/chelators, and a road map was proposed for zinc trafficking in the developing barley grain based on laser capture microdissection and gene expression profiling [35,36,37]. The small MW ligands of Zn represented by the phytochelatins and phytochelatin synthetases are present, respectively, at low amount and mRNA expression level in the embryo and endosperm, but relatively consistent in the transfer cells and aleurone [35]. Furthermore, P and zinc contents inversely correlate [29], and other low MW ligands, such as nicotianamine (NA), are present at low concentrations in mature grain. Increasing the amount of NA in mature grains is possible by transgenic approaches, such as overexpressing nicotianamine synthase (NAS) isogenes [38,39] or T-DNA activated NAS2, which ultimately increased the bioavailable zinc bound to NA [40]. Metallothioneins are small cysteine-rich metal-binding proteins that present in the cytoplasm of plant cells, and thought to bind zinc in large amounts, as they regulate the zinc homeostasis in the cytosol depending on the zinc demand, oxidative stresses, or detoxification in the case of hyperaccumulating plants [41,42,43]. Barley metallothioneins are a multigene cytosolic family (10 isoforms) involved in zinc homeostasis, of which in barley, MT3 and MT4 are the only isoforms localized in the grain aleurone layer, possessing differential zinc binding capabilities [25]. However, upon zinc fertilization or starvation, their expression level does not change. Instead, some of the isoforms are downregulated. Another study shows that barley MTs do not detoxify excess Zn and Cd, but possibly excess Cu [44].

Bioavailable Zn is bound to low molecular weight compounds, such as Zn–nicotianamine, whereas insoluble Zn is bound to high MW proteins [45]. It has been clarified that low MW N-ligand compounds represent less than 10% of the total bioavailable zinc in the grains. The majority of the sink ligand (>90%) is still bound to unknown subcellular proteins inside the storage vacuoles of the cells belonging to both the aleurone layer and embryo [46].

We investigated, here, whether proteins can chelate zinc as an alternative to low molecular weight ligands. The zinc storage proteins (ZSPs) of barley grain, which represented our zinc sink candidates, were isolated by a combination of tissue microdissection, Osborne fractionation, SDS-PAGE, PVDF blotting, dithizone DTZ (diphenyl thiocarbazone, DTZ) staining, and zinc ion affinity chromatography (Zn-IMAC) followed by protein identification by nanoscale liquid chromatography coupled to tandem mass spectrometry (nanoLC MS/MS). We identified the most abundant zinc binding proteins in the embryo and aleurone layers (7S globulins, late abundant embryogenesis proteins, dehydrins, many isoforms of non-specific lipid transfer proteins, and alpha amylase trypsin inhibitors), and in the endosperm (B-hordeins, and by smaller extent gamma- and D-hordeins). We propose that these abundant zinc binding proteins might represent a pool of potential zinc storage proteins in the developing barley grain.

## 2. Materials and Methods

### 2.1. Plant Material

Barley grains (*Hordeum vulgare* cv. Golden Promise) were germinated and cultivated as described before [47]. Developing grains (harvested at 30 days after pollination, DAP) were surface sterilized with 2% hypochlorite solution for 5 min, and followed by extensive washing with sterile water before microdissection. Four main tissue types were isolated: (1) grain seed coats; (2) aleurone and subaleurone layers; (3) embryo; and (4) endosperm.

### 2.2. Osborne Protein Fractionation of the Microdissected Barley Tissues

A modified Osborne fractionation protocol [8,48] was used with five consecutive extraction steps, without or with DTT pre-treatment. Two hundred milligrams of tissue were used from each tissue type. All the solutions contained protein inhibitor cocktail (0.4% protease and 0.4% of phosphatase inhibitors, Sigma P8849 and P0044, respectively). The plant material was homogenized in extraction buffer in liquid nitrogen using mortar and pestle. The albumins were extracted with an acidic extraction buffer (25 mM acetate buffer pH 4.5) at 1:10 (*v*/*w*) ratio. After centrifugation at 10,000 *g* for 2 min at 4 °C, the supernatant containing the albumins was concentrated and dialyzed against the same extraction buffer by Vivaspin 500 centrifugal devices (GE-Healthcare, Uppsala, Sweden), and stored at 4 °C until further analysis, while the pellet was used for the isolation of the globulins. The pellet was resuspended in an extraction buffer containing salt (100 mM Tris-HCl pH 7.5, 500 mM NaCl), and vortexed at 400 rpm for five cycles of 1 min. Between the vortexing steps, the samples were kept for 2 min on ice. The protein mixture was centrifuged at 20,000 *g* for 5 min at 4 °C. The supernatant containing the globulins was concentrated and dialyzed using the same extraction buffer, and stored at 4 °C until further analysis, while the pellet was used for the next step, the hordein extraction. The hordeins were extracted with a propanol extraction buffer (100 mM acetate buffer pH 4.5, containing 55% 2-propanol). The hordeins were extracted by vortexing at room temperature, followed by centrifugation at 20,000 *g* for 5 min. The supernatant was concentrated by dialyzing against 55% 2-propanol, and stored at 4 °C until analysis. Finally, the glutelin-containing protein fraction was obtained by treating the pellet of the hordein isolation with an alkaline buffer (100 mM ethanolamine buffer pH 11, 1% 2-mercaptoethanol, and 1% SDS). The glutelin-enriched fractions were also dialyzed using Vivaspin centrifugal devices against 25 mM Tris-HCl pH 8.0 containing 1 mM DTT, and stored at 4 °C until analysis.

For the DTT treatment, 25 mM DTT was added to each of the isolated Osborne protein fractions (except for the hordein fractions, where 50 mM was used), and the samples were incubated for 2 h at 4 °C, followed dialysis using Vivaspin 500 centrifugal devices (GE-Healthcare, Uppsala, Sweden). For each fraction, the same extraction buffer was used as dialysis buffer, as described above, but without protease and phosphatase inhibitors.

### 2.3. SDS-PAGE, Coomassie, Zinc Blotting, and DTZ Staining Assay

Proteins were resolved using a XCell SureLock™ Electrophoresis cell (Invitrogen, Waltham, MA, USA) on 4–12% NuPAGE Bis-Tris gel (Invitrogen) with MOPS SDS running buffer (Invitrogen) at constant 150 V for 1–1.5 h. The SDS-PAGE gel was stained either with Coomassie blue or silver staining procedures.

For Coomassie blue staining, the gel was first fixed in fixing solution (50% methanol and 10% glacial acetic acid) for 1 h with gentle agitation. Thereafter, the gel was stained for 1–2 h with staining solution containing 0.1% Coomassie Brilliant Blue R-250, 50% methanol and 10% glacial acetic acid. Destaining was done by washing the gel in a solution containing 20% methanol and 5% glacial acetic acid. After staining, images were taken using BioRad GelDoc™ (Bio-Rad Laboratories, Copenhagen, Denmark) or Epson perfection V500 photo scanner (Epson, Herlev, Denmark).

The protein gels were blotted on polyvinylidene fluoride (PVDF) membrane, and the zinc binding protein were visualized by dithizone staining, according to Uddin and co-workers [49].

### 2.4. Zn-IMAC Chromatography of Osborne Fractionated Proteins of the Microdissected Barley Tissues

Two rounds of zinc affinity chromatography were performed. In the first round, all solutions in both the Osborne fractionation and Zn-IMAC chromatography experiments contained inhibitor cocktails (0.4% protease and 0.4% of phosphatase inhibitors, Sigma-Aldrich Denmark A/S, Brøndby, Denmark, cat. Num. P8849 and P0044, respectively). For the second round of Zn-IMAC chromatography, the Osborne fractionated proteins were DTT pre-treated, and no protease and phosphatase inhibitors were added for the zinc affinity step.

PureProteome™ Nickel Magnetic Beads (250 μL, Merck, Darmstadt, Germany) were stripped with 50 mM Tris-HCl buffer pH 7.5, 100 mM EDTA, 500 mM NaCl, washed with 100 mM acetate buffer pH 5.0, and finally with MilliQ water. The beads were magnetically captured, and the liquid removed between the steps by using magnetic stand. The stripped PureProteome™ Magnetic Beads were loaded with zinc in a solution of 20 mM Tris-HCl pH 8.0 containing 100 mM ZnSO_4_ for 2 min. Multiple washing steps using 20 mM Tris-HCl pH 8.0 were performed to remove the excess zinc. The efficiency of washing of the non-bonded zinc had been monitored by dithizone staining of the washing fluids. The zinc magnetic beads equilibrated in 250 μL 20 mM Tris-HCl pH 8.0 were mixed with 100 μg proteins from each tissue for the zinc capture chromatography. A washing step was optimized for each Osborne fraction. The washing step for the albumins and globulins was performed in 20 mM Tris-HCl pH 8.0, 0.1% Rapigest (Waters, Milford, MA, USA), 150 mM NaCl, and 20 mM imidazole. For the hordeins and glutelins, the washing step consisted of the above washing buffer made in 55% 2-propanol. Three washing steps were performed in each case. The captured zinc binding proteins bound to the magnetic beads were processed directly for the proteomic analysis.

Osborne protein fractions pre-treated with DTT, as described above, were also subjected to the Zn-IMAC capture step (second Zn-IMAC capture step), and the captured proteins on the beads were processed directly for MS/MS identification. The second Zn-IMAC capture step was quantitative, as all samples contained 50 fmol/μL of yeast enolase 1 (UniProt accession number P00924) as a standard.

The maltose binding protein (MBP5, NEB, New England Biolab by Bionordika Denmark A/S, Herlev, Denmark) and alcohol dehydrogenase 1 (yeast ADH1, UniProt P00330), have been chosen as negative and positive controls for the Zn-IMAC chromatography [49]. In brief, 20 μL Zn-IMAC magnetic beads were mixed with 1 μg control proteins, respectively (maltose binding protein (MBP5, NEB cat. N. E8046) or alcohol dehydrogenase 1 (ADH1, Sigma-Aldrich cat. N. A7011), and equilibrated in 250 μL 20 mM Tris-HCl pH 8.0. The excess of proteins was washed away with three wash steps performed in 20 mM Tris-HCl pH 8.0, 0.1% Rapigest (Waters, Milford, MA, USA), 150 mM NaCl, and 20 mM imidazole. Hence, the beads with immobilized ligands were subjected to trypsin digestion, and the peptides identified by nanoLC-MS/MS.

### 2.5. Sample Preparation for Proteomic and MS Analysis

Proteins captured on beads were denatured, and cysteines reduced in 50 μL of 0.1 M ABC buffer (ammonium bicarbonate buffer pH 8.0, 0.1% Rapigest, 40 mM DTT) at 56 °C for 45 min. Cysteine alkylation was performed adding 10 μL of 500 mM iodoacetamide (IAM), and the mixture was incubated for 45 min at room temperature in brown Eppendorf tubes. The excess of IAM was quenched with DTT (80 mM final concentration). The beads were harvested by magnetic stand, and the buffer was exchanged with 100 μL of 0.1 M ABC buffer containing 0.1% Rapigest. The bead-captured alkylated proteins were digested with 250 ng of proteomic grade trypsin (Pierce, Thermoscientific, Rockford, IL, USA) in the same buffer. The digestion was performed at 37 °C overnight with moderate shaking, and stopped by adding 5 μL of 5% formic acid (FA). Peptide desalting was performed by Supel-Tips C18 micropipette tips, according to the manufacturer specifications (Supelco by Sigma-Aldrich Denmark A/S, Copenhagen, Denmark). The desalted peptides were dried at 35 °C overnight (Speedvac Christ RVC 2-18, Germany), and dissolved in 0.1% FA containing 3% acetonitrile (can). Prior to loading onto the nanoLC, the desalted peptides were passed through 0.22 μm centrifugal filters (Durapore^®^-PVDF, Merck Millipore, Darmstadt, Germany). The amount of peptide per sample was evaluated by 280 nm ABS using a Nanodrop 1000 (Thermoscientific, Pittsburgh, PA, USA). Per each sample, 500 ng of desalted peptides were analyzed in triplicate onto an Xbridge BEH130 C18 5 μm desalting/trap column with a BEH300 C18 1.7 μm nanoUPLC analytical capillary column (100 μm × 100 mm) on an Acquity nano UPLC-LC system interfaced with a nano source to a Q-TOF Premiere MS (Waters, Milford, MA, USA). The entire length of the LC run was 90 min, starting with a gradient of acetonitrile in 0.1 FA (0 to 40%) from 0 to 70 min, followed by 95% acetonitrile wash. Data acquisition was performed in V positive mode. The acquisition method was either the data-independent acquisition (DIA) by all ion fragmentation (MSe), or data dependent (DDA). MS and MS/MS data recorded in DDA mode (MS scan every 1 s, MS/MS every 1 s with selection of 4–8 ions and real-time mass exclusion) and MSe mode were performed by Masslynx version 4.1 (Waters). MSe runs were analyzed by the Protein Lynx Global Server software ver. 2.5 (Waters, Milford, MA, USA) using Leu-Enk (leucine-enkephaline) lock mass standard with 556.2771 *m/z* for 30 ppm error tolerance for MS data and 30 ppm error for MSe data. Search parameters included semi-trypsin as standard protease. The fixed modification was the carbamidomethyl cysteine (∆Mass + 57.02), and other variable modifications: deamination NQ (+0.98), oxidation M,H,W (15.99), dehydration DSTY,C-term (−18.01), hydroxylation D, K, N, P, R, Y (+15.99), pyrrolidinone P (−30.01), pyroglutamic P (13.98), and pyro-Glu from Q (−17.03), and pyro-Glu from E (−18.01). For absolute and relative quantification, yeast enolase 1 (UniProt accession number P00924) tryptic digest (Waters, MassPREP Enolase Digestion Standard, cat. #186002325) was spiked as 50 fmol/μL (final concentration) into each sample. This label-free quantification was performed according to the Protein Lynx Global Server (PLGS) ver 2.5 by spectral counting and Hi3 algorithm (Waters, Milford, MA, USA). The label-free quantification method is based on the relationship between MS signal response and protein concentration: the average MS signal response for the three most intense tryptic peptides per mole of protein is constant within a coefficient of variation of less than 10%. Given an internal standard, this relationship is used to calculate a universal signal response factor. The universal signal response factor (counts/mol) is shown to be the same for all proteins tested [50]. The label-free quantification method comprehensively reduces tens of thousands of ion detections to a simple inventory list of peptide precursors along with their time-resolved fragment ions [51]. The spectral counting for protein quantification is known as label-free protein quantitation using weighted spectral counting [52]. Waters has implemented the APEX algorithm, which calculates Absolute Protein EXpression levels based on learned correction factors, MS/MS spectral counts, and each protein’s probability of correct identification [53]. The MSe data acquisition, in fact, has been supplied by a known spiked trypsin digest at known protein concentration, and another algorithm Hi3 (based on the High abundant 3 peptides for each protein, including the spiked ones) has converted the spectral counting into an absolute amount. In our experiment data acquisition, LCMSE mode was used in the presence (spike) of known tryptic digested protein (50 fmol/μL). A decoy search with false discovery rate of 4 was chosen as default method. In both modes, a ppm error max of 20 +/+ 5 ppm in survey mode (MS1) was ensured by the calibration peptide Leu-Enk (leucine-enkephaline, lock mass) which was recorded every 60 seconds as fragmented spectra, and MS/MS had a maximum of 30 ppm as error. All the acquisitions were performed in triplicate, but the software used (PLGS v2.5 or PEAKS studio v.6) was unable to provide peptide statistics, i.e., ANOVA (*p* value).

### 2.6. Light Microscopy

Developing grains at 30 DAP were fixed in 60% ethanol and 10% glycerol for 3 h before DTZ staining. The fixed grains were cut manually into half, longitudinally, from which 15 μm thin sections were produced by a cryostat microtome (MICROM HM550, Thermo Fisher Scientific, Roskilde, Denmark), as described before [35]. DTZ staining was performed by 45 s exposure to 1 mM DTZ in 50% methanol in 50 mM acetate buffer pH 4.5. Thereafter, the sections were neutralized by 0.5 M ABC buffer in 50% methanol, and mounted in Histo-clear mounting reagent, before microscopy. Pictures were taken in bright field mode using a Zeiss Axioplan II microscope (Carl Zeiss A/S, Birkerød, Denmark).

### 2.7. Gel Fractionaction on Gelfree 8100 and Silver Staining

For separation of protein by molecular weight in liquid phase recovery GELFREE^®^ 8100 fractionation system (Expedeon/Protein Discovery Inc., San Diego, CA, USA) was used according to the manufacturer’s protocol. In short, 150 μL protein sample was mixed with 40 μL 5× loading buffer and 10 μL 1 M DTT, and heated for 100 °C for 10 min. Then, 200 μL of sample was loaded into the chamber for separation using the 10% Tris-acetate cartridge (Expedeon Expedeon/Protein Discovery Inc., San Diego, CA, USA) with the mass range 3.5–100 kDa, and resolution between 15 and 100 kDa. The samples were loaded in different loading chambers, and run in different channels so that there was no carryover or cross contamination. Samples were collected from the electroelution chamber, 180 μL each, at established time points over 2–3 h (see Supplementary Figure S1 for details on time points). SDS-PAGE was carried out in XCell SureLock™ Electrophoresis cell (Invitrogen by Thermo Fisher Scientific, Roskilde, Denmark) on 4–12% NuPAGE Bis-Tris gel with MOPS SDS running buffer at constant 120 V for 1–1.5 h. Afterwards, SDS-PAGE gels were fixed in 50% methanol and 1% acetic acid, and stained by silver staining using the PageSilver™ Silver Staining Kit (Fermentas by Thermo Fisher Scientific, Roskilde, Denmark), according to the manufacturer’s procedure.

## 3. Results

### 3.1. Manual Microdissection of Barley Developing Grain

A manual microdissection was performed followed by dithizone (DTZ) staining. Dithizone is known to have a preferential pink to red staining color development upon nanomolar to micromolar zinc chelation [54]. At the stage of 30 DAP, the seed coat and the embryo removal were easy to perform without a microscope, but the aleurone/subaleurone layers were peeled off by micro tweezers and scalpel under a microscope. The collected microdissected grain tissues were divided into four groups: (1) seed coats; (2) aleurone and subaleurone; (3) embryo; and (4) endosperm, which could contain traces of aleurone and subaleurone as possible contaminant.

### 3.2. DTZ Staining of the Longitudinal Section of Half Barley Grain

**Figure 1:**
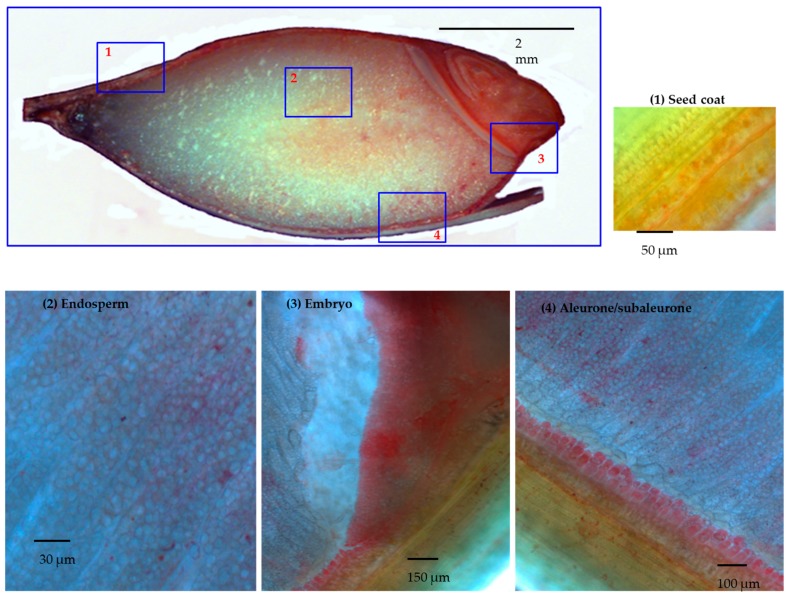
Longitudinal section of half barley grain stained by dithizone (DTZ) and further magnification of selected tissues. Panel **1**: seed coat; Panel **2**: the endosperm; Panel **3**: embryo; Panel **4**: aleurone/subaleurone layers.

Upon mild fixing conditions, described in Materials and Methods, 30 DAP barley grains had been stained with DTZ as half grain cut longitudinally. For deeper analysis, a series of 15 μm thin sections were stained from selected tissues as well (Figure 1). The distribution of the DTZ was highly biased toward the embryo and the aleurone layers. Upon magnification of the endosperm, regions surrounding the starch cells showed DTZ staining as well. The dilution effect in the endosperm could be due to the abundance of starch compared to the proteins.

The main picture: 30 DAP barley grain cut longitudinally and DTZ stained. Different magnifications of the 15 μm thick slices of selected tissue: in panel 1, in the green seed coats, the photosynthesis was active and DTZ signal was present; in panel 2, in the endosperm, the DTZ signal seems diluted, due to granule starch abundance. Higher DTZ signal was hence detected respectively in the embryo (panel 3) and in the aleurone/subaleurone layers (panel 4).

### 3.3. Osborne Protein Fractionation of the Microdissected Barley Tissues, Zn-IMAC Capture, and nanoLC MS/MS Identification

Proteins from the four microdissected groups (1) grain coats; (2) aleurone and subaleurone; (3) embryo; and (4) endosperm were subjected to a modified Osborne fractionation. On each of the four microdissected tissues, four Osborne fractions (albumins, globulins, hordeins, and glutelins) were obtained. The proteins of these Osborne fractions of each microdissected group were resolved on SDS-PAGE, and blotted onto PVDF membranes before DTZ staining, except for the fractions of the aleurone/subaleurone layers, where we had material only for the second round Zn-IMAC.

The first round of Zn-IMAC enrichment was performed with the DTZ positive preselected Osborne protein fractions without DTT pre-treatment. Zn-IMAC captured proteins were identified by qualitative MS.

The quantitative MS study for identifying the second round Zn-IMAC captured proteins (DTT treated) was achieved by spiking yeast enolase 1 into each tryptic digest of the samples (50 fmol/μL). The maltose binding protein (MBP5, NEB) and the alcohol dehydrogenase 1 (yeast ADH1, P00330), were tested as negative and positive controls, respectively. As expected, the yeast ADH1 was effectively captured while the negative control MBP5 was not isolated by the metal ion affinity chromatography. The typical binding capacity of PureProteome™ Nickel Magnetic Bead is 1–5.5 μg of recombinant protein per μL of bead suspension, in case of HIS6 tagged proteins. Using 20 μL of the original Merck suspension beads will ensure from 20 to 110 μg of polyhistidine-tagged proteins. In our system, we did not used nickel, but zinc, and alcohol dehydrogenase 1 has been our golden standard. In case of alcohol dehydrogenase 1, it has been recovered with a yield of about 12% on the beads. The absolute recovery has been 0.62 ng/μL, which multiplied for 200 μL (total resuspension volume for the beads and the MS sample) gives 124 ng that, as compared to 1 μg used, gives a total efficiency of binding of 12.4%.

The major abundant zinc binding proteins that were captured by Zn-IMAC without (first round) or with DTT pre-treatment (second round) were identified by MS/MS. The top protein candidates with zinc binding capacity from each of the microdissected fractions are presented according to their abundance and protein groups in Supplementary Table S1 (without DTT pre-treatment, and ordered in a descending way by spectral counting abundance coefficient, and the PLGS score (not shown)) and in Supplementary Table S2 (with DTT pre-treatment, ordered in a descending way by the absolute quantification obtained by spiking yeast enolase 1 as described in Material and Methods).

#### 3.3.1. Seed Coat (Group 1)

**Figure 2:**
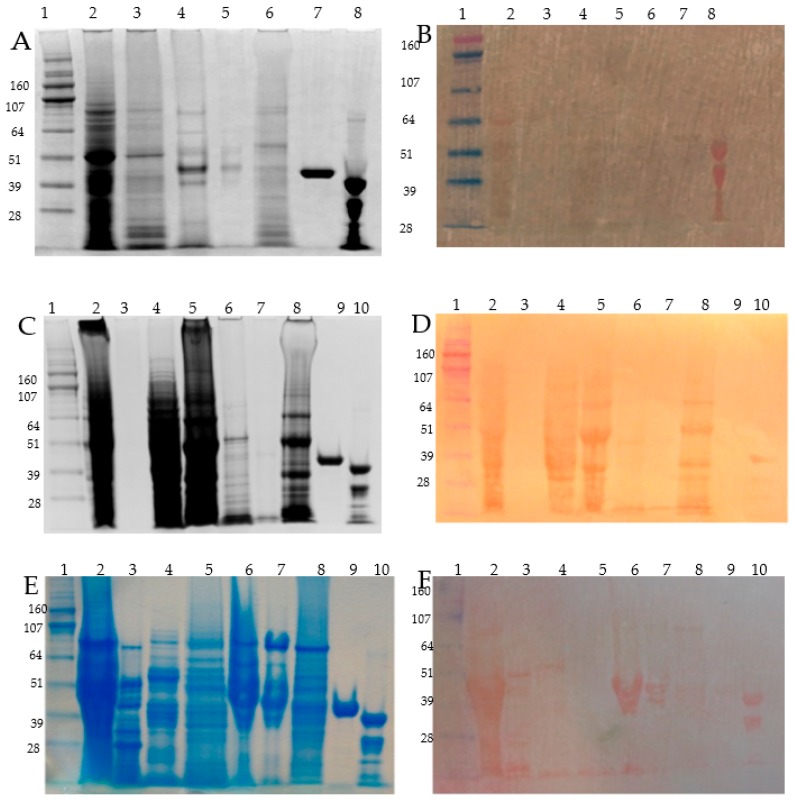
SDS-PAGE of Osborne fractionated barley grain proteins and related PVDF/DTZ zinc blot. Panel **A**, **C** and **E**: SDS-PAGE (4–12% NuPAGE) separation of the protein fractions, Panel **B**, **D** and **F**: zinc overlayed PVDF membrane blotted proteins and DTZ staining. Panel **A** and **B**: seed coats, Panerl **C** and **D**: embryo; Panel **E** and **F**: a endosperm proteins. Description of lanes: Panel **A** and **B**: (1) HiMark Pre-stained High Molecular Weight Protein Standard (Invitrogen) (MW in kDa); (2) albumins; (3) globulins; (4) alcohol soluble proteins extracted without DTT; (5) alcohol soluble proteins extracted with DTT; (6) glutelin; (7) maltose binding protein (MBP5, NEB UK); (8) alcohol dehydrogenase 1 (ADH1, Sigma-Aldrich) showing a partial proteolysis band in this particular batch; Panel **C** and **D**: (1) Hi-Mark Pre-stained marker (MW in kDa); (2) total extract in SDS-sample buffer; (3) albumin < 5 kDa; (4) concentrated albumins, (5) globulins; (6) alcohol soluble proteins extracted with DTT; (7) alcohol soluble proteins extracted with DTT; (8) glutelins; (9) maltose binding protein (MBP5, NEB England); (10) alcohol dehydrogenase 1 (ADH1, Sigma-Aldrich); Panel **E** and **F**: (1) HiMark Pre-stained High Molecular Weight Protein Standard, (Invitrogen) (MW in kDa); (2) endosperm total extract with SDS-sample buffer; (3) alcohol soluble fraction from embryo; (4) endosperm albumins; (5) endosperm globulins; (6) endosperm prolamin/hordein fraction extracted without DTT; (7) endosperm prolamin/hordein fraction extracted with DTT; (8) endosperm glutelin; (9) maltose binding protein (MBP5, NEB England); (10) alcohol dehydrogenase 1 (ADH1, Sigma-Aldrich,) showing a partial proteolysis band in this particular batch.

Among the four protein fractions, only the albumin showed Zn binding with DTZ staining (Figure 2). The detected protein had an estimated MW of 50 kD (Figure 2B). The seed coats of the 30 DAP grains were green, which suggested active photosynthesis and the possible presence of chloroplast proteins, but the contamination with aleurone proteins cannot be excluded. Qualitative analysis was performed for the albumin fractions after the first capture step, and the proteins were listed according to descending spectral counting, hence accounting for their semi-quantitative relative abundance. According to the MS identification, the most abundant protein corresponded to the large chain of ribulose bisphosphate carboxylase (Rubisco; P05698) (Supplementary Table S1). Quantitative analysis was performed after the second capture step on the albumin and globulin fraction. These two fractions were selected because of their higher abundance in proteins, despite that only the albumin fractions showed DTZ positive bands (Figure 2B). The other abundant proteins were the translation elongation factor EFTu-EF1A (F2DAU4), and the chloroplastic glyceraldehyde-3-phosphate dehydrogenase A (F2D714) (Supplementary Table S2).

#### 3.3.2. Aleurone/Subaleurone (Group 2)

During manual microdissection, the aleurone/subaleurone tissue peeled off from the seed coats easily, but the endosperm had to be removed with a scalpel. Therefore aleurone/subaleurone tissue (group 2) could contain contamination from the endosperm, but was supposed to be enriched with aleurone/subaleurone proteins. Due to the limited amount of material recovered after Osborne fractionation, DTZ staining was not performed. We have performed a quantitative Zn-IMAC affinity chromatography step and MS identification on the fractions of albumins, globulins, and prolamins. The most abundant zinc binding proteins for both the albumins and the globulins were non-specific lipid transfer proteins (P07597, P20145, M0VYA0), alpha amylase/trypsin inhibitors (F2EEH7, P01086, P11643, P1369, P32936, P01086, O24000), and hordothionins (Q9FS19, P01545, P21742). Among the prolamins, the most abundant proteins were B3-hordeins (I6TEV5), followed by gamma-1 hordeins (I6SJ17), B1-hordein (Q3YAF9), gamma 3 hordein (I6TEV2), D-hordein (F2EA67), and alpha amylase inhibitor (O24000) CMd3 (Supplementary Table S2).

#### 3.3.3. Embryo (Group 3)

In the embryo, the major Zn binding proteins were present in the albumin, globulin, and glutelin Osborne fractions, according to the DTZ staining (Figure 2C,D). Prolamin extracted without or with DTT did not show any DTZ positive band, indicating very low contamination from the endosperm. A first round qualitative Zn-IMAC screening was performed for embryo albumins and globulins, and identified by MS/MS (Supplementary Table S1). In the albumins, the highest presence of proteins, judged by spectral counting, were represented by the late embryogenesis abundant proteins (LEA isoforms: F2EKY2, M0ZDL8), the non-specific lipid transfer protein (Q5UNP2, F2CY84), and the Bowman Birk type trypsin inhibitor (M0Y075). The globulin fraction was dominated by the 7S embryo globulin (Q03678), followed by LEA proteins (F2EKY2, A9Q2Q5), elongation factors (Q6LAA4, F2CWX1), ricinB (lectin_isoform 2, F2DIC8), globulin 2 (F2EBM4, vicilin-like 7S storage proteins), oleosins (F2E8X4, Q43769, Q43770), and a lot of ribosomal 40S and 60S subunit proteins (F2CT73, F2DIR3, F2D483, F2D4A4, etc.). After DTT pre-treatment, the second Zn-IMAC step revealed the quantitative presence of thionins, grain softness proteins, Kunitz type subtilisin-chymotrypsin inhibitors, and late embryogenesis abundant proteins (LEA5) in the albumin fraction (Supplementary Table S2). In the globulin fraction, the most abundant proteins were members of the cupin superfamily, of which the 7S globulins (M0XUU4 and M0XH58) and the embryo globulin (Q03678) were the main representatives (Supplementary Table S2). After dialysis and DTT pre-treatment, from the quantitative Zn-IMAC procedure, some of the candidate proteins present in the first screening were missing, e.g., the dehydrin type 6 (Supplementary Tables S1 and S2).

#### 3.3.4. Endosperm (Group 4)

In the endosperm, all Osborne fractions were positive for the presence of zinc, according to the DTZ staining. Low molecular weight DTZ positive bands were detectable in all the protein fractions, while in the globulin and prolamin fractions, the presence of other DTZ stained proteins were detected as well (Figure 2D,F). For the first qualitative Zn-IMAC step, we have analyzed the strongest DTZ positive Osborne fractions: globulins and prolamins (Supplementary Table S1). According to their abundance, judged by their spectral counting, the zinc binding proteins captured in the globulin fraction were hordothionins (P01545, P21742), D-hordein (I6SW34), amylase/trypsin inhibitors (M0ULY1, P28041), globulin 7S isoform (M0XH58), gamma-thionin (M0Y046), beta amylase (P16098), and non-specific lipid transfer proteins (M0VYA0, P20145). In the prolamin fraction, the captured abundant proteins were D hordein (I6SW34), B3 hordeins (P06471, I6QP72), alpha hordothionin (P01545), and gamma-1 hordein (P17990).

The effect of free cysteines in chelating zinc was tested during the second round of Zn-IMAC in the albumin, globulin, and prolamin fractions (Supplementary Table S2). In the endosperm albumins, the most abundant zinc binding proteins were amylase/trypsin inhibitors (O24000, P11643, P01086, E7BB45, E7BB45, P32936, P13691, Q546U1), non-specific lipid transfer protein (F2EE76), and in lower amounts, alpha hordothionin (P01545), hordoindoline a (Q5URW5), and grain softness protein (A9E4R4). In the globulin fraction, the zinc binding proteins were in the order of abundance, as follows: alpha amylase inhibitors (O24000, E7BB45), thionin (F2EE63), and D hordein (Q40054). Finally, in the prolamins fraction, the most abundant captured zinc binding proteins were the B3 hordeins (P06471, Q2XQF1), gamma 2 hordein (Q70IB4), followed by B1-hordeins (I6QP72) and D-hordeins (I6SW34).

Overall, the quantitative Zn-IMAC capture analysis revealed the predominant presence of the following dominant captured proteins in barley grain at 30 DAP: B3-hordein (1668 ng/g FW), embryo globulin (976 ng/gr FW), Rubisco large subunit (830 ng/g FW), globulin 1S (524 ng/g FW), non-specific lipid transfer protein 1 (106 ng/g FW), alpha amylase trypsin inhibitor CMd (164 ng/g FW), alpha amylase trypsin inhibitor CMd3 (106 ng/g FW), and thionins (84 ng/g FW).

### 3.4. Detection of the Most Abundant Zn-IMAC Captured Proteins after DTT Pre-Treatment

Metallothioneins contains zinc as cofactors, and zinc storage can be accomplished by sulfhydryl group chelation, i.e., metallothioneins, or as for zinc finger transcription factor, it could be due to the mixed chelation mechanism represented by both sulfhydryl groups and histidines. During the first-round capture (without DTT), the capture steps relied on the reducing cysteines present on those proteins in the native proteins just after the extraction and prior further processing. Redox-sensitive cysteine residues are present in many zinc binding proteins, and cysteine oxidation might impair both the function and the zinc binding capabilities of the proteins [55]. A DTT pre-treatment followed by dialysis has been used to further enrich the protein fractions for zinc binding proteins, by reducing their cysteines prior the second-round zinc affinity purification (Supplementary Table S2). Among the most abundant Zn-IMAC captured proteins were embryo globulin (Q03678), oleosin (Q43769), late embryogenesis abundant (LEA) proteins (F2EKY2 and A9Q2Q5), the ribosomal protein S7e (F2EG94), transcription elongation factor 2 (F2CWX1), the mitochondrial import inner membrane translocase subunit Tim17/22/T23 (F2CVG5), B3 hordein (P06471), dehydrin 6 (Q9SPA6, F2DZZ0), and elongation factors (F2CWX1, F2DAU4). In Supplementary Table S3 are presented the peptide mapping and peptide MM-MS details.

### 3.5. Protein Size Fractionation and Electro-Elution by Gelfree 8100, Zinc DTZ Blotting, and MS/MS Identification of the High DTZ Stained Bands

Selected Osborne protein extracts were further size separated by 1D-PAGE using Gelfree 8100 fractionation station (Expedeon Inc., San Diego, CA, USA) for size fractionation and collection (electro-elution). In total, 15 sub-fractions were electro-eluted for the embryo globulins and run onto a separate SDS-PAGE, and after blotting the gel onto PVDF membrane, the blotted proteins were zinc overlayed, washed, and DTZ stained as described before [49], as shown in Supplementary Figure S1. These preparative electro-eluted fractions were further analyzed by shotgun MS/MS identification, and scored by spectral counting based on the PEAKS ver 6.0-10lgP parameter (Bioinformatics Solutions Inc., Waterloo, ON, Canada). The protein bands showing the highest level of zinc binding intensity were also cut from the blotting paper and subjected to individual MS/MS identification, as shown in Supplementary Table S4. The main pink-red bands on the PVDF membranes had three main different apparent MWs recorded after DTZ staining (Supplementary Figure S1), but after excision and tryptic peptide MS/MS identification, all three gave almost the same proteins IDs (Supplementary Table S4). This is not surprising, since globulins undergone N- and C-terminal processing by proteolysis (i.e., removal of leader peptide or pro-region N-/C-terminal) as is known for wheat Glo-3 [56].

## 4. Discussion

The majority of proteomic studies on plant have been performed with *Arabidopsis* and rice leaves under diverse abiotic stresses, imbalances in mineral nutrition, and enhanced concentrations of heavy metals [57]. Furthermore, the recent proteomic approaches concentrate on the molecular mechanisms and metabolic activities required for heavy metal hyperaccumulation and detoxification in specialized groups of plant [57,58,59,60]. The current zinc related knowledge is more comprehensive on transcriptome analyses of transporters [35], and on a restricted group of low molecular weight organic acids [61], phytochelatins [33], deoxymugineic acid [62], and nicotianamides [63] chelating zinc. Among the cereals to date, a proteome analysis and identification of the zinc binding proteins has been only performed in wheat grains [64]. Our study represents the first comprehensive proteomic analysis of zinc binding proteins in developing barley grains.

Zinc is considered a “borderline” metal, as it does not have a strong preference for coordinating with either oxygen, nitrogen, or sulfur atoms [65,66]. In proteins, Zn usually shows tetrahedral coordination by four ligands, such as sulfur from cysteine, nitrogen from histidine, oxygen from aspartate and glutamate, and to much lesser extent, the hydroxyl of tyrosine, and the carbonyl oxygen of either asparagine or glutamine [65,66]. The major association with S or O atoms in the aleurone layers of the grain is suggested [27], and recently, speciation of Zn in wheat grain has been investigated [64]. As a matter of fact, the first zinc binding proteome of the wheat grain obtained using state of the art metal detection techniques has been reported [64]. Comparing their results to ours, it is clear that zinc is present in many metabolic enzymes, and late embryogenesis abundant proteins, but especially in gamma-gliadins/hordeins, as well as 7S/12S globulins both in wheat and barley ([64] Supporting Information Tables S5 and S6).

Overall, our two capture procedures with or without DTT pre-treatment yielded many enriched Zn-IMAC captured protein candidates in the four protein groups of the Osborne fractions, and demonstrated a minor effect of the involvement of the disulfide groups. On the other hand, our procedure could have left out proteins in which zinc was already tightly bound, and as such, the saturation prevented the binding of extra zinc ions (e.g., metallothioneins). Dialysis or lowering pH to 4.5 is not enough to remove zinc from metallothioneins; only oxidants or nitric oxide can liberate zinc from them (i.e., from ZmMT4 or long time incubation in concentrated mineral acid solutions [67].

Our Zn-IMAC system setup was consistently reliable to isolate Zn binding proteins according to the positive (ADH1) and negative controls (MBP5) used in the experiments. The yeast enolase 1 protein digest has been spiked in all the samples to a fixed concentration (50 fmol/μL), in order to get label-free quantification, according to the PLGS spectral counting and Hi3 algorithm in the second round of Zn-IMAC experiments. Our results indicated that the members of cupin superfamily, followed by the prolamin superfamily, could have a key role in zinc binding and possible storage in the protein storage vacuole of embryo and aleurone/subaleurone layers.

### 4.1. Zn Binding Proteins in Globulins

The cupins are a functionally diverse superfamily of proteins that share two short conserved consensus sequence motifs, [G(X)5HXH(X)11G] and [G(X)5P(X)4H(X)3N], where X could be any amino acid residue, and represents the metal binding site in many, but not all members of the superfamily [68]. The X-ray crystal structure of some cupins showed that the enzyme’s active site contains essential zinc ions [68]. From plants, the best characterized cupins are germins (single chain cupins) and globulins (bicupins) [69]. The germin-like protein gene family functions to confer broad-spectrum disease resistance [70], while globulins are seed storage proteins [71]. Based on the sedimentation coefficient, globulins are divided into the 11S legumin-type globulins and 7S vicilin-type globulins [72]. The globulins are known to form trimers (7S) or hexamers (11S) of monomers that have also undergone posttranslational proteolytic formation of two subunits (acid and basic chains) linked by disulfide bonds. Besides the globulin orthologue of soybean, the beta glycinin (GLYG1_SOYBN) is known already to interact with zinc and zinc-phytate [73,74]. The glycinin apoprotein divested of zinc and the zinc reconstituted proteins have been analyzed by ultracentrifugal analysis, and the zinc was able to shift the Svedberg coefficient from 11.1S (apoprotein) to 13.1S (plus zinc) [73]. Besides, β-conglycinin (7S) and glycinin (11S) are known to be the major storage globulins in soybean seed, and both precursors and proteolytic mature forms have been detected as the major copper binding proteins after Cu^2+^-IMAC [75]. Furthermore, β-conglycinin can be specifically purified by zinc-IMAC [76].

Assessed by label-free quantification, the embryo globulin (Q03678) emerged as the most abundant protein, followed by other 7S globulins isoforms, Globulin-1S_a and b (M0XUU4, M0XH58) isoforms among the zinc-IMAC captured cupins, but the opposite abundance pattern was observed after the second round of Zn-IMAC (Supplementary Tables S1 and S2). In our modified Osborne fractionation, the initial shift of pH coupled to salt wash might have removed zinc from the barley cupins, and allowed the cupins to be captured effectively. The cupins possess several clusters of aspartate and histidine as in embryo globulin (e.g., in the N-terminal), but after the peptide mapping for those aspartate/histidine clustered regions we did not detect any peptides according to our MS analysis (Supplementary Table S3). Similar results were reported for the soybean beta-conglycinin alpha subunit [77]. Recently, a similar zinc-IMAC technique was used to capture peptide hydrolysate from sesame proteins, and found that 7S and 11S cupin peptides and lipid transfer proteins were effectively captured, supporting our findings here [78]. Despite 11S globlulins genes/proteins existing in barley (UniProt F2E9N0/ M0Z4S0), 11S globulins had not been quantitatively detected in high amount at 30 DAP in barley grains, and in any of the fractions analyzed, mainly 7S globulins were present (Supplementary Tables S1 and S2). This result was in accordance with previous findings [79]. The 7S globulins possess high glutamic acid and histidine contents, and targeted to the protein storage vacuole.

### 4.2. Zinc Binding Proteins in Prolamin Fractions

Increased Zn concentration in the mature grain is reported to be correlated with B-hordein content after Zn fertilization in barley [16]. From the zinc-IMAC captured prolamin members, the B3-hordein (P06471 and Q2XQF1) was quantitatively the most abundant hordein, followed by D-hordein (I6SW34) and γ-hordeins (P17990 and Q70IB4). Other zinc-IMAC captured members of the prolamin fraction (alcohol soluble proteins) included many isoforms of the alpha amylase/trypsin inhibitors, non-specific lipid transfer proteins, thionins (thionins, hordothionin), and indolines (grain softness protein, hordoindolines). Thionins can be involved, along with metallothioneins in the mechanism of redox regulation of zinc transport, and contribute, in the cytosol, to zinc homeostasis or the response to pathogen attack [80,81].

It was suggested that structural genes of prolamin of *Triticeae*, the 2S globulins of castor bean and rape, the CM proteins of barley and wheat, and some inhibitors of proteases and α-amylases are evolved from a single ancestral gene encoding a protease inhibitor with a single domain structure [82,83,84]. From crystallographic studies, it was reported that pumpkin trypsin inhibitor, CMTI-I (ITR1_CUCMA) is capable of binding zinc in a tetrahedral and symmetric fashion through glutamic acid residues [85]. Furthermore, an earlier study with ^65^Zn application at anthesis in bread wheat (*Triticum aestivum*) also showed Zn incorporation in different Osborne fractions, such as glutenin (47–65%), albumin and globulins (9–20%), gliadins (1–3%), and remaining proteins (9–20%) [86]. A possible involvement of rice prolamins in zinc binding has been indicated [19], and the increase of the overall protein content is positively correlated with a higher Zn content in wheat whole grain and in the endosperm [17,87].

### 4.3. Other Groups of Zn Binding Proteins

The other zinc-captured proteins were ribosomal proteins, proteinase inhibitors, dehydrins, the late embryogenesis abundant (LEA) proteins, Rubisco large subunit, elongation factors, transcription factors, and other proteins (Supplementary Tables S1 and S2).

We were able to capture many ribosomal proteins by the zinc-IMAC techniques, confirming the previous findings that bacterial orthologues can be isolated the same way [88]. The high propensity for zinc ribbon structures in the ribosome is intriguing, and the Zn^2+^ ions in these proteins stabilize the structures of the small globular domains in which they are found, rather than binding to RNA themselves [89]. Many ribosomal proteins have been shown to be zinc captured, and their binding does not seem to be regulated by reduced cysteine, since after DTT pre-treatment, only 40S ribosomal protein S28-like seems to be affected (Supplementary Table S2). Eukaryotic ribosomal L24, L36, L37, L37a, and L44 proteins possess a GATA-type zinc finger [90,91]. From our zinc binding experiments, many other ribosomal proteins were able to bind tightly to the Zn-IMAC (Supplementary Tables S1 and S2), but despite being captured, their mechanism of zinc binding via zinc motifs remains to be identified.

Some of serine protease inhibitors can recruit and incorporate physiological zinc at the active site to mediate potent, selective inhibition [92]. As for Kunitz, the subtilisin and chymotrypsin inhibitors do not contain zinc, but these inhibitors are known to coordinate zinc in the active site of metalloproteases (e.g., subtilisin, carboxypeptidase, etc.) during the formation of the enzyme–inhibitor complexes [93].

Another group of proteins involved in metal detoxification/antioxidant effects and water desiccation/stress are the dehydrins [94] and the late embryogenesis abundant (LEA) proteins [95]. Dehydrins and LEA have known zinc binding capabilities, even if they also bind other metals (i.e., calcium, iron, etc.) and they are also implicated in cold and desiccation tolerance [96]. In dehydrins, the metal binding is regulated by phosphorylation [97]. Barley possess 13 dehydrin (Dhn from 1 to 13) genes of which most of them respond to water stress, others to cold stress (Dhn5, Dhn 8, Dhn 11) having a very different ABA sensitivity [98]. Dehydrin proteins are characterized by conserved motifs: Y-sequence (DEYGNP), S-sequence, a 12 serine rich disordered loop, and Lys-rich sequence motif, the K-sequences (EKKGVMEKIKEKLPG). The combination/permutation of these conserved motifs divides Dhn in 4 sub classes of isoforms: Y_n_SK_n_, SK_n_, K_n_, and KS, respectively. Barley dehydrin type 6 (Dhn6, Q9ZTR5) has a Y_2_SK_3_ structure, its zinc binding properties found here, or its implication in drought stress, but not in cold tolerance [98], cannot be possible to extrapolate either by its primary structure or domains. However, barley Dhn6 shares some sequence similarities with *Arabidopsis* ERD14, which is phosphorylated in some serines of such motifs, enhancing the binding of calcium, but also zinc, the latter being a competitive inhibitor of calcium [99]. Some LEA proteins, which are not plant specific, belong to the dehydrin superfamily, since some of them possess the K-sequence. LEA are classified based on their amino acid composition and motifs [100] that lead to high hydrophilicity (measured by a GRAVY, Grand Average of Hydropathy index) and heat stability in solution. In *Arabidopsis*, 9 LEA groups have been identified based on specific motifs [100]. LEA are also called intrinsically disordered protein (IDP), since they possess an intrinsically disordered domain (IDD) which becomes folded upon dehydration, where the ion binding capability is highest [101]. In barley, the LEA_4 group F2EKY2 and A9Q2Q5 have a predicted localization respectively in the chloroplast and the secretion pathway, probably the Protein Storage Vacuole (PSV), according to TargetP server [102], while the rest are predicted to have cytosolic localization. The precise physiological role of LEA proteins is not known, and the distinct groups are suggested to function differently: LEA_1 to have water-binding capacity in the cytosol; LEA_2 and/or dehydrins stabilizing cellular structures; LEA_3 protective of mitochondrial membranes against dehydration damage; LEA_4 sequestering ions and replacing water molecules in mitochondrial/chloroplast and vacuoles [103]. Reversible phosphorylation of proteins is an important mechanism also involved in the acquisition of desiccation tolerance, and in the resurrection plant *Craterostigma plantagineum*, LEA_2 proteins have been found phosphorylated during such acquisitions [104]. In our experiments, the late embryogenesis abundant proteins, (LEA) F2EKY2 and A9Q2Q5, were captured only in the first round of Zn-IMAC capture step, indicating a possible speculative involvement of phosphorylation as a zinc binding mechanism.

Rubisco large subunit is not known as a zinc binding protein, nevertheless, it has affinity for both CO_2_ and micromolar Mg^2+^ ion [105,106] and hence, zinc could act as a tightly binding ligand during Zn-IMAC purification.

A discrete number of barley elongation factors, eEF1 (Q6LAA4, F2DAU4) and eEF2 (F2CWX1), were captured as well. Eukaryotic elongation factors possess known zinc ribbon C-terminal domains needed for RNA recognition [107]. Initiation factors are known to possess a zinc finger domain as the captured barley eIF4A1 (F2D2Z0, Supplementary Table S1) [108]. The transcription factor AP2 type (APETALA2, B8K2C3) was the only abundant transcription factor detected (Supplementary Table S2) [109].

## 5. Conclusions

The aim of the study was hence to investigate whether zinc can be bond to proteins as an alternative to low molecular weight ligands in the barley grain. We have enriched and identified, by Zn-IMAC and MS/MS analysis, many potential candidate proteins that could be in vivo zinc ligands and storage sink proteins. In this study is reported the first proteome identification of the most abundant zinc binding proteins in developing barley grains. We have also quantified the most abundant protein captured by Zn-IMAC method, since we believed that the candidate binding proteins for storage should also be present in larger amount. To summarize, in the embryo dominates the 7S embryo globulin (Q03678) and a leaf specific thionin (lemma thionin, C9W327). Furthermore, embryo specific expression was found for the Bowman–Birk inhibitor (M0Y075). In the endosperm, the major globulin detected was also a globulin 7S type (M0XH58), followed by B-hordeins (P06471, Q2XQF1), and gamma-hordein (I6SJ17, I6TEV2), whereas the alpha and beta hordothionins (P01545, P21742 and F2EE63) are shared with the aleurone layer, but the gamma-thionin (M0Y046) is unique for the endosperm. Aleurone, scutellar epithelium, scutellar provascular strands, and the outermost embryonic leaves contained the highest concentration of dehydrins [110]. However, Dehydrin 6 (Q9ZTR5) was the only dehydrin isoform captured by zinc chromatography in the embryo, possibly reflecting its abundance in this tissue. Regarding the tissue specificity, analogously, the late embryogenesis abundant (LEA) group (M0ZDL8, F2EKY2, A9Q2Q5, F2ECH4, H2E688, Q05190) detected in our Zn-IMAC screen were only mainly captured in the embryo, despite that their expression is both bi-specific in the aleurone and embryo. We propose that the most abundant zinc binding proteins, the 7S globulins and members of the prolamin superfamily, as well as B/γ-hordeins, LEA and dehydrins, could have important roles in zinc binding, and be involved maybe in the storage of other mineral(s)/metabolite(s). These proteins could be relevant for future zinc biofortification strategies in cereals, but further validation is required, in particular, their metal binding specificities in vitro and in vivo. On the other hand, the captured zinc binding proteins should be validated for other heavy metal binding specificities, as not only zinc, but other bivalent metals could be capable of binding to these proteins (e.g., calcium, magnesium, iron, cadmium, etc.). Future research, hence, is needed to clone and express such zinc storage proteins as recombinant forms, in order to validate their in vitro metal binding ability, and the stoichiometry of metal molecules, e.g., zinc able to be stored. Finally, a definitive proof of concept would be their ectopic transgenic expression, in other non-sink tissues (i.e., leaves/roots) to verify their in vivo sink action.

## Supplementary Materials

The following are available online www.mdpi.com/2227-7382/6/1/3/s1, Table S1: MS/MS identification of the zinc-IMAC captured proteins related to selected Osborne fractionated proteins originated from the group 1 (seed coats plus aleurone), group 3 (embryo) and the group 4 (endosperm) micro-dissected tissues. Table S2: MS/MS identification of the zinc-IMAC captured proteins using a DTT pre-treatment and related to selected Osborne fractionated proteins coming from the most DTZ positive micro-dissected tissues. Table S3: Peptide mapping of the most abundant Zn-IMAC captured proteins. Figure S1: SDS-PAGE of embryo globulins size separated (and electroeluted) by Gelfree 8100 and related DTZ stain after zinc overlay. Table S4: Total protein composition of a selected electroeluted protein fraction from embryo globulins.
