# Supplementary material for: Enrichment and Identification of the Most Abundant Zinc Binding Proteins in Developing Barley Grains by Zinc-IMAC Capture and Nano LC-MS/MS
Source: Proteomes. 2018 Jan 17;6(1):3. doi: 10.3390/proteomes6010003 (PMC5874762; doi:10.3390/proteomes6010003)
Supplement: Supplementary file 1 [file proteomes-06-00003-s001.zip › Supplementary files Article Dionisio final.docx]

Article

**Enrichment and identification of the most abundant zinc binding proteins in developing barley grains by Zinc-IMAC capture and nano LC-MS/MS**

**Giuseppe Dionisio ^1,^*^,†^, Mohammad Nasir Uddin^2,†^ and Eva Vincze ^3,^***

SUPPLEMENTAL TABLES

TABLE OF CONTENTS:

**Table S1:** First round of MS/MS identification of captured zinc binding proteins for each microdissected fractions presented according to their four protein groups. …………………….. 2

**Table S2:** Second-round of MS/MS identification of captured zinc binding proteins for each microdissected fractions presented according to their four protein groups. ……………..……… 5

**Table S3.** Peptide mapping of the most abundant Zn-IMAC captured proteins (excel file)

**Figure S1.** SDS-PAGE of embryo globulins size separated (and electroeluted) by Gelfree 8100 and related DTZ stain after zinc overlay………………..……………………………………….……10

**Table S4.** Total protein composition of a selected electroeluted protein fraction from embryo globulins. ………………………………………………………………………………………….…… 11

**Table S1.** **First round of MS/MS identification of captured zinc binding proteins for each microdissected fractions presented according to their four protein groups.** The protein extraction has been performed without the use of DTT. The proteins have been ordered in a descending way based on their tryptic peptides spectral counting abundance.

| **Accession** | **ID** | **Description** | **MW (Da)** | **pI (pH)** | **Coverage (%)** | **Peptides** |
| --- | --- | --- | --- | --- | --- | --- |
| **Seed coats (group 1) albumins** | |  |  |  |  |  |
| P05698 | RBL_HORVU | Ribulose bisphosphate carboxylase large chain | 53044 | 6.22 | 20.04 | 12 |
| M0VYA0 | M0VYA0_HORVD | Type 1 Non Specific Lipid Transfer Protein Precursor | 12321 | 9.54 | 25.41 | 5 |
| P20145 | NLTP2_HORVU | Probable non specific lipid transfer protein | 10349 | 6.94 | 37.25 | 5 |
| A1E9I8 | ATPA_HORVU | ATP synthase subunit alpha chloroplastic | 55260 | 6.32 | 14.48 | 5 |
| F2CV78 | F2CV78_HORVD | Non specific lipid transfer protein | 9200 | 8.99 | 28.57 | 4 |
| P01545 | THNA_HORVU | Alpha hordothionin | 13587 | 6.17 | 18.11 | 4 |
| Q40004 | RBS_HORVU | Ribulose bisphosphate carboxylase small chain chloroplastic | 19407 | 8.94 | 27.01 | 3 |
| **Embryo (group 3) albumins** | |  |  |  |  |  |
| M0ZDL8 | M0ZDL8_HORVD | Late embryogenesis abundant protein | 27486 | 5.56 | 31.16 | 18 |
| F2EKY2 | F2EKY2_HORVD | Late embryogenesis abundant protein | 70517 | 7.34 | 32.95 | 14 |
| M0Y075 | M0Y075_HORVD | Bowman Birk type trypsin inhibitor | 19176 | 8.02 | 42.46 | 14 |
| Q5UNP2 | Q5UNP2_HORVD | Non specific lipid transfer protein 2 | 12354 | 8.96 | 41.94 | 12 |
| M0WCQ8 | M0WCQ8_HORVD | Peptidyl-prolyl cis-trans isomerase | 17354 | 8.72 | 32.92 | 11 |
| F2CY84 | F2CY84_HORVD | Non specific lipid transfer protein | 12332 | 8.51 | 42.98 | 10 |
| H2E688 | H2E688_HORVU | Late embryogenesis abundant protein | 9955 | 7.34 | 40.86 | 9 |
| Q05190 | LE19A_HORVU | Late embryogenesis abundant protein B19 1A | 9955 | 7.34 | 59.14 | 8 |
| P52572 | REHY_HORVU | 1 Cys peroxiredoxin PER1 | 23948 | 6.34 | 31.65 | 8 |
| M0VYA0 | M0VYA0_HORVD | Type 1 Non Specific Lipid Transfer Protein Precursor | 12321 | 9.54 | 25.41 | 6 |
| F2CY84 | F2CY84_HORVD | Non specific lipid transfer protein | 12332 | 8.51 | 33.06 | 5 |
| M0UXX1 | M0UXX1_HORVD | Acetyl-CoA acetyltransferase, cytosolic | 38080 | 5.83 | 10.84 | 5 |
| Q42848 | Q42848_HORVU | Non specific lipid transfer protein | 12321 | 9.54 | 25.41 | 4 |
| A3RHQ9 | A3RHQ9_HORVD | Glyceraldehyde 3 phosphate dehydrogenase | 26273 | 6.62 | 10.61 | 3 |
| P20145 | NLTP2_HORVU | Non specific lipid transfer protein 2 | 10349 | 6.94 | 12.75 | 2 |
| M0VN21 | M0VN21_HORVD | Subtilisin-chymotrypsin inhibitor-2A | 7961 | 5.06 | 20.27 | 2 |
| Q9M4C9 | Q9M4C9_HORVU | Subtilisin-chymotrypsin inhibitor-2A (bci 7), potato inhibitor like | 7913 | 5.09 | 17.57 | 1 |
| **Embryo (group 3) globulins** | |  |  |  |  |  |
| Q03678 | Q03678_HORVU | Embryo globulin | 72209 | 6.83 | 44.58 | 77 |
| F2EKY2 | F2EKY2_HORVD | Late Embryogenesis Abundant (LEA) protein | 70517 | 7.34 | 40.20 | 51 |
| F2DIX5 | F2DIX5_HORVD | ATP-dependent DNA helicase 2 subunit ku80 | 79263 | 5.78 | 31.10 | 42 |
| F2CT73 | F2CT73_HORVD | Ribosomal protein L2_A | 28173 | 11.49 | 50.19 | 33 |
| F2CWX1 | F2CWX1_HORVD | Elongation factor 2 | 85087 | 5.72 | 28.44 | 31 |
| F2DIR3 | F2DIR3_HORVD | Ribosomal protein S4e | 29931 | 10.56 | 43.77 | 28 |
| F2D483 | F2D483_HORVD | 40S ribosomal protein S8 | 25248 | 10.90 | 53.33 | 27 |
| F2DIC8 | F2DIC8_HORVD | RicinB_lectin_2 | 38713 | 6.29 | 54.18 | 26 |
| F2EBM4 | F2EBM4_HORVD | Globulin 2, Cupin, Vicilin-like (7S plant seed storage proteins) | 76876 | 5.48 | 29.36 | 22 |
| F2D4A4 | F2D4A4_HORVD | 60S ribosomal protein L4 | 44149 | 11.01 | 49.75 | 21 |
| F2D5B2 | F2D5B2_HORVD | ATP synthase subunit beta | 59320 | 5.80 | 33.03 | 20 |
| Q43769 | Q43769_HORVU | Oleosin | 18482 | 10.02 | 87.50 | 18 |
| F2CVG5 | F2CVG5_HORVD | mitochondrial import inner membrane translocase subunit Tim17/22/T23 | 18731 | 7.51 | 47.78 | 18 |
| F2DSU6 | F2DSU6_HORVD | Guanine nucleotide-binding protein beta subunit-like protein | 36141 | 5.96 | 47.29 | 17 |
| Q6LAA4 | Q6LAA4_HORVU | Elongation factor 1 alpha | 49137 | 9.48 | 24.83 | 16 |
| F2D0U3 | F2D0U3_HORVD | Ribosomal protein S19 | 17392 | 10.71 | 44.16 | 14 |
| F2EEU3 | F2EEU3_HORVD | Ribosomal protein S17_B | 17804 | 11.00 | 35.22 | 13 |
| F2D9E0 | F2D9E0_HORVD | Ribosomal protein L10e | 27580 | 11.55 | 34.43 | 12 |
| F2D2Z0 | F2D2Z0_HORVD | Eukaryotic initiation factor 4A-1 | 46914 | 5.15 | 32.61 | 12 |
| Q43770 | Q43770_HORVU | Oleosin | 16020 | 9.59 | 29.11 | 11 |
| F2EG94 | F2EG94_HORVD | Ribosomal protein S7e | 22146 | 10.13 | 48.96 | 11 |
| F2D861 | F2D861_HORVD | Ribosomal protein S12/S23 | 15650 | 10.91 | 42.25 | 10 |
| F2CVF1 | F2CVF1_HORVD | Ribosomal protein S15_S13e_A | 17115 | 10.96 | 32.45 | 10 |
| F2CV33 | F2CV33_HORVD | Ribosomal protein L14 | 15005 | 10.93 | 39.29 | 10 |
| F2DNP8 | F2DNP8_HORVD | 60S ribosomal protein L36 | 12522 | 11.86 | 34.23 | 9 |
| F2CW48 | F2CW48_HORVD | Ribosomal protein S25 | 12060 | 11.03 | 44.44 | 9 |
| F2CSK4 | F2CSK4_HORVD | Glucose and ribitol dehydrogenase-like protein | 37492 | 9.41 | 27.38 | 9 |
| F2CSC5 | F2CSC5_HORVD | Ribosomal protein L19 | 24214 | 11.86 | 16.75 | 8 |
| Q39999 | Q39999_HORVU | Gamma thionin | 8925 | 7.80 | 74.39 | 7 |
| F2EJZ0 | F2EJZ0_HORVD | Ribosomal protein L32e | 15544 | 11.11 | 23.31 | 7 |
| F2DT01 | F2DT01_HORVD | Ribosomal protein L14 | 14701 | 11.08 | 45.32 | 7 |
| F2DVU2 | F2DVU2_HORVD | 60S ribosomal protein L13 | 24114 | 11.35 | 24.52 | 7 |
| F2D0I7 | F2D0I7_HORVD | Ribosomal protein L2 | 24735 | 10.65 | 21.92 | 6 |
| F2EFR1 | F2EFR1_HORVD | 40S ribosomal protein S6 | 28401 | 11.10 | 18.80 | 6 |
| F2CUI5 | F2CUI5_HORVD | Ribosomal protein L24e | 18388 | 11.10 | 19.75 | 6 |
| F2CUE9 | F2CUE9_HORVD | Glyceraldehyde-3-phosphate dehydrogenase (GAPDH) | 36490 | 6.78 | 28.19 | 6 |
| F2EKP2 | F2EKP2_HORVD | Ribosomal_S30 | 6908 | 12.43 | 17.74 | 5 |
| Q9M4C9 | Q9M4C9_HORVU | Subtilisin-chymotrypsin inhibitor bci-7 , potato inhibitor I like | 7913 | 5.09 | 72.97 | 5 |
| F2E8X4 | F2E8X4_HORVD | Oleosin | 16934 | 7.93 | 14.37 | 5 |
| Q9ZTR5 | Q9ZTR5_HORVU | Dehydrin 6 | 47622 | 8.66 | 18.13 | 5 |
| F2EAU8 | F2EAU8_HORVD | 60S ribosomal protein L26-1 | 17532 | 11.54 | 24.20 | 5 |
| F2CV88 | F2CV88_HORVD | Ribosomal_S26e | 15682 | 11.44 | 27.08 | 4 |
| F2CZ65 | F2CZ65_HORVD | Ribosomal protein L28e | 16248 | 10.73 | 27.27 | 4 |
| F2DB00 | F2DB00_HORVD | Prohibitin-1, mitochondrial-like | 30578 | 6.14 | 19.30 | 4 |
| D0F044 | D0F044_HORVU | Calmodulin Fragment | 13235 | 3.98 | 31.90 | 3 |
| F2CUP7 | F2CUP7_HORVD | 60S ribosomal protein L4-1 | 8908 | 10.34 | 32.50 | 3 |
| F2DUY9 | F2DUY9_HORVD | Ribosomal protein S19/S15 | 17166 | 10.50 | 26.80 | 2 |
| **Endosperm (group 4) globulins** | |  |  |  |  |  |
| P01545 | THNA_HORVU | Alpha hordothionin | 13587 | 6.17 | 46.46 | 29 |
| P21742 | THNB_HORVU | Beta hordothionin | 14592 | 6.71 | 35.29 | 20 |
| I6SW34 | I6SW34_HORVD | D hordein | 79303 | 7.96 | 10.98 | 13 |
| M0ULY1 | M0ULY1_HORVD | Alpha amylase trypsin inhibitor CMb | 15806 | 5.13 | 54.55 | 11 |
| M0XH58 | M0XH58_HORVD | Globulin-1 S2 isoform | 46337 | 6.60 | 25.42 | 9 |
| P28041 | IAAA_HORVU | Alpha amylase trypsin inhibitor CMa | 15489 | 5.84 | 33.10 | 7 |
| M0Y046 | M0Y046_HORVD | Gamma-thionin | 8925 | 7.80 | 37.80 | 6 |
| M0VYA0 | M0VYA0_HORVD | Type 1 Non Specific Lipid Transfer Protein Precursor | 12321 | 9.54 | 25.41 | 6 |
| P16098 | AMYB_HORVU | Beta amylase | 59609 | 5.50 | 17.76 | 6 |
| M0XDM7 | M0XDM7_HORVD | RicinB lectin 2 | 26044 | 6.11 | 19.67 | 5 |
| F2DQB4 | F2DQB4_HORVD | Histone H2B | 15095 | 10.55 | 18.84 | 4 |
| P20145 | NLTP2_HORVU | Non specific lipid transfer protein | 10349 | 6.94 | 26.47 | 3 |
| P06471 | HOR3_HORVU | B3 hordein | 30176 | 7.53 | 9.85 | 3 |
| E7BB45 | E7BB45_HORVD | Barley trypsin inhibitor CMc | 11241 | 7.44 | 21.15 | 1 |
| **Endosperm (group 4) hordeins** | |  |  |  |  |  |
| I6SW34 | I6SW34_HORVD | D hordein | 79303 | 7.96 | 11.51 | 43 |
| P06471 | HOR3_HORVU | B3 hordein | 30176 | 7.53 | 9.85 | 22 |
| I6QP72 | I6QP72_HORVD | B hordein | 33481 | 7.06 | 7.59 | 13 |
| P01545 | THNA_HORVU | Alpha hordothionin | 13587 | 6.17 | 28.35 | 9 |
| P17990 | HOG1_HORVU | Gamma hordein 1 | 34714 | 7.95 | 11.80 | 8 |

**Table S2.** **Second-round of MS/MS identification of captured zinc binding proteins for each microdissected fractions presented according to their four protein groups.** The protein extraction has been performed with the use of DTT. The proteins have been ordered in a descending way based on their absolute quantification of their tryptic peptides.

| **Accession** | **ID** | **Description** | **MW (Da)** | **pI (pH)** | **Peptides** | **Coverage (%)** | **Amount (fmol)** | **Amount (ngrams)** |
| --- | --- | --- | --- | --- | --- | --- | --- | --- |
| **Seed Coats (group 1) Albumins** | |  |  |  |  |  |  |  |
| Q7YMR9 | Q7YMR9_HORVS | Ribulose bisphosphate carboxylase large chain | 53044 | 6.22 | 21 | 50.10 | 78.23 | 4.15 |
| P00924 | ENO1_YEAST | Enolase 1 EC 4 2 1 11 | 46642 | 6.15 | 73 | 37.39 | 50.00 | 2.34 |
| F2EE76 | F2EE76_HORVD | Non specific lipid transfer protein | 11827 | 8.30 | 4 | 31.78 | 23.40 | 0.28 |
| F2D6I8 | F2D6I8_HORVD | Glyceraldehyde-3-phosphate dehydrogenase (GAPDH) | 36588 | 6.75 | 13 | 34.72 | 21.30 | 0.78 |
| F2DAU4 | F2DAU4_HORVD | Translation elongation factor EFTu-EF1A | 62011 | 7.40 | 15 | 32.14 | 13.10 | 0.81 |
| F2EEH7 | F2EEH7_HORVD | Trypsin alpha amylase inhibitor | 11751 | 8.30 | 9 | 47.66 | 11.73 | 0.14 |
| F2D714 | F2D714_HORVD | Glyceraldehyde-3-phosphate dehydrogenase A, chloroplastic | 42672 | 7.66 | 8 | 13.47 | 11.50 | 0.49 |
| M0VFR1 | M0VFR1_HORVD | LRR receptor-like serine/threonine-protein kinase RFK1 | 55251 | 7.85 | 4 | 10.02 | 10.01 | 0.56 |
| F2DY47 | F2DY47_HORVD | DNA directed RNA polymerase | 133636 | 7.94 | 5 | 1.68 | 5.96 | 0.81 |
| P11643 | IAAD_HORVU | Alpha amylase trypsin inhibitor CMd | 18513 | 6.07 | 4 | 37.43 | 5.87 | 0.11 |
| P32936 | IAAB_HORVU | Alpha amylase trypsin inhibitor CMb | 16514 | 5.67 | 7 | 28.86 | 3.63 | 0.06 |
| M0Z0D3 | M0Z0D3_HORVD | Malate dehydrogenase | 33153 | 5.33 | 3 | 14.89 | 3.13 | 0.10 |
| P01086 | IAAE_HORVU | Alpha amylase trypsin inhibitor CMe | 16124 | 7.42 | 3 | 17.57 | 2.92 | 0.05 |
| M0VDC1 | M0VDC1_HORVD | SF-assemblin/beta giardin homologue | 66115 | 10.19 | 5 | 6.81 | 2.49 | 0.17 |
| M0VFV9 | M0VFV9_HORVD | Alpha amylase trypsin inhibitor CMc | 15724 | 4.94 | 3 | 23.29 | 1.85 | 0.03 |
| **Seed Coats (group 1) Globulins** | |  |  |  |  |  |  |  |
| P00924 | ENO1_YEAST | Enolase 1 EC 4 2 1 11 | 46642 | 6.15 | 64 | 40.83 | 50.00 | 2.34 |
| F2CUP3 | F2CUP3_HORVD | Histone H4 | 11402 | 11.88 | 6 | 29.13 | 6.35 | 0.07 |
| M0VYA0 | M0VYA0_HORVD | Type 1 Non Specific Lipid Transfer Protein Precursor | 12321 | 9.54 | 13 | 41.80 | 5.47 | 0.07 |
| P32936 | IAAB_HORVU | Alpha amylase trypsin inhibitor CMb | 16514 | 5.67 | 6 | 28.86 | 1.25 | 0.02 |
| **Aleurone/Subaleurone (group 2) Albumins** | |  |  |  |  |  |  |  |
| P00924 | ENO1_YEAST | Enolase 1 EC 4 2 1 11 | 46642 | 6.15 | 51 | 42.20 | 50.00 | 2.34 |
| P07597 | NLTP1_HORVU | Non specific lipid transfer protein 1 | 12293 | 8.31 | 17 | 54.70 | 41.18 | 0.53 |
| F2EEH7 | F2EEH7_HORVD | Trypsin alpha amylase inhibitor | 11751 | 8.30 | 8 | 62.62 | 18.81 | 0.23 |
| P01086 | IAAE_HORVU | Alpha amylase trypsin inhibitor CMe | 16124 | 7.42 | 11 | 18.24 | 15.57 | 0.26 |
| P20145 | NLTP2_HORVU | Non specific lipid transfer protein | 10349 | 6.94 | 5 | 23.53 | 15.34 | 0.17 |
| P11643 | IAAD_HORVU | Alpha amylase trypsin inhibitor CMd | 18513 | 6.07 | 7 | 53.22 | 14.06 | 0.27 |
| P13691 | IAA2_HORVU | Alpha amylase inhibitor BDAI 1 | 16417 | 5.19 | 6 | 37.50 | 10.76 | 0.18 |
| P32936 | IAAB_HORVU | Alpha amylase trypsin inhibitor CMb | 16514 | 5.67 | 8 | 39.60 | 7.69 | 0.13 |
| M0ZDP1 | M0ZDP1_HORVD | Bifunctional nuclease | 23979 | 8.40 | 6 | 7.76 | 7.30 | 0.18 |
| P16968 | IAA1_HORVU | Alpha amylase inhibitor BMAI 1 | 15805 | 5.15 | 4 | 26.71 | 3.81 | 0.06 |
| P28041 | IAAA_HORVU | Alpha amylase trypsin inhibitor CMa | 15489 | 5.84 | 4 | 44.83 | 2.44 | 0.04 |
| Q9FSI9 | HINB1_HORVU | Hordoindoline B1 | 16110 | 8.30 | 9 | 23.13 | 2.38 | 0.04 |
| **Aleurone/Subaleurone (group 2) Globulins** | |  |  |  |  |  |  |  |
| P00924 | ENO1_YEAST | Enolase 1 EC 4 2 1 11 | 46642 | 6.15 | 57 | 41.06 | 50.00 | 2.34 |
| P11643 | IAAD_HORVU | Alpha amylase trypsin inhibitor CMd | 18513 | 6.07 | 18 | 58.48 | 42.63 | 0.82 |
| P01545 | THNA_HORVU | Alpha hordothionin | 13587 | 6.17 | 17 | 39.37 | 38.52 | 0.56 |
| M0WPU2 | M0WPU2_HORVD | Chromosome segregation ATPases | 231298 | 4.83 | 27 | 11.10 | 30.23 | 7.03 |
| P32936 | IAAB_HORVU | Alpha amylase trypsin inhibitor CMb | 16514 | 5.67 | 12 | 39.60 | 27.84 | 0.48 |
| P13691 | IAA2_HORVU | Alpha amylase inhibitor BDAI 1 | 16417 | 5.19 | 11 | 47.37 | 24.41 | 0.42 |
| P01086 | IAAE_HORVU | Alpha amylase trypsin inhibitor CMe | 16124 | 7.42 | 13 | 23.65 | 15.90 | 0.27 |
| P21742 | THNB_HORVU | Beta hordothionin | 14592 | 6.71 | 15 | 31.62 | 13.73 | 0.21 |
| E7BB45 | E7BB45_HORVD | Barley trypsin inhibitor CMc | 11241 | 7.44 | 7 | 56.73 | 10.23 | 0.12 |
| P28041 | IAAA_HORVU | Alpha amylase trypsin inhibitor CMa | 15489 | 5.84 | 7 | 35.86 | 8.32 | 0.13 |
| M0VYA0 | M0VYA0_HORVD | Type 1 Non Specific Lipid Transfer Protein Precursor | 12321 | 9.54 | 10 | 46.72 | 4.83 | 0.06 |
| P34951 | IAAC_HORVU | Alpha amylase trypsin inhibitor CMc | 15168 | 6.78 | 8 | 41.26 | 3.83 | 0.06 |
| I6SW34 | I6SW34_HORVD | D hordein | 79303 | 7.96 | 3 | 5.35 | 3.58 | 0.29 |
| Q9FSI9 | HINB1_HORVU | Hordoindoline B1 | 16110 | 8.30 | 6 | 24.49 | 2.78 | 0.05 |
| P16968 | IAA1_HORVU | Alpha amylase inhibitor BMAI 1 | 15805 | 5.15 | 4 | 26.71 | 2.69 | 0.04 |
| P07596 | IAAS_HORVU | Alpha amylase subtilisin inhibitor | 22149 | 7.88 | 11 | 46.31 | 2.09 | 0.05 |
| M0WYN5 | M0WYN5_HORVD | Transposase (WRKY-GCM1 zinc finger) | 227943 | 4.47 | 17 | 6.28 | 0.62 | 0.14 |
| **Aleurone/Subaleurone (group 2) Hordeins** | |  |  |  |  |  |  |  |
| I6TEV5 | I6TEV5_HORVU | B3 hordein | 35440 | 7.51 | 24 | 21.29 | 102.17 | 3.67 |
| P00924 | ENO1_YEAST | Enolase 1 | 46787 | 6.15 | 75 | 47.83 | 50.00 | 2.34 |
| I6SJ17 | I6SJ17_HORVD | Gamma 1 hordein | 34714 | 7.95 | 18 | 23.61 | 38.08 | 1.34 |
| E7BB45 | E7BB45_HORVD | Barley trypsin inhibitor CMc | 11241 | 7.44 | 15 | 75.96 | 29.14 | 0.34 |
| Q3YAF9 | Q3YAF9_HORVD | B hordein | 34442 | 8.68 | 4 | 14.00 | 16.84 | 0.59 |
| Q5URW5 | Q5URW5_HORVD | Hordoindoline a | 16472 | 8.13 | 10 | 32.21 | 13.54 | 0.23 |
| I6TEV2 | I6TEV2_HORVU | Gamma 3 hordein | 33763 | 6.70 | 3 | 5.42 | 12.14 | 0.42 |
| F2EE63 | F2EE63_HORVD | Thionin | 14562 | 6.71 | 13 | 50.00 | 11.63 | 0.18 |
| G8FGD4 | G8FGD4_HORVU | Limit dextrinase inhibitor | 15962 | 6.93 | 10 | 51.70 | 11.63 | 0.17 |
| F2EA67 | F2EA67_HORVD | D-Hordein | 79273 | 7.78 | 11 | 10.98 | 10.60 | 0.92 |
| O24000 | O24000_HORVU | Alpha amylase subtilisin inhibitor CMd3 | 18458 | 7.68 | 8 | 52.05 | 6.77 | 0.13 |
| F2EI31 | F2EI31_HORVD | Dimeric alpha-amylase inhibitor (Bdai-1) | 16605 | 5.19 | 9 | 71.43 | 6.67 | 0.12 |
| F2E235 | F2E235_HORVD | Embryogenesis (PGG domain) transmembrane protein-like | 48338 | 6.65 | 10 | 27.15 | 3.63 | 0.18 |
| F2DHS6 | F2DHS6_HORVD | Serine/threonine CCR4-like cytokinin-regulated kinase | 91450 | 7.82 | 9 | 17.10 | 1.75 | 0.16 |
| Q84VT9 | Q84VT9_HORVU | Trypsin inhibitor | 16076 | 6.80 | 3 | 8.84 | 1.53 | 0.03 |
| F2EFF9 | F2EFF9_HORVD | FIZZY-RELATED 3-like cell cycle switch protein | 52128 | 9.75 | 8 | 17.28 | 0.45 | 0.02 |
| Q8S409 | Q8S409_HORVD | Hordoindoline a | 15648 | 7.95 | 9 | 33.80 | 0.43 | 0.01 |
| G1UH41 | G1UH41_HORVS | Hordoindoline b1 | 16110 | 8.30 | 10 | 30.61 | 0.42 | 0.01 |
| **Embryo Albumins** | |  |  |  |  |  |  |  |
| P00924 | ENO1_YEAST | Enolase 1 | 46787 | 6.15 | 58 | 37.07 | 50.00 | 2.34 |
| Q9M4C9 | Q9M4C9_HORVU | Subtilisin-chymotrypsin inhibitor bci-7 | 7913 | 5.09 | 12 | 60.81 | 33.10 | 0.26 |
| C9W327 | C9W327_HORVD | Thionin | 14611 | 7.21 | 9 | 36.50 | 26.99 | 0.42 |
| B8K2C3 | B8K2C3_HORVD | Transcription factor AP2 type (APETALA2 or EREBP) | 14865 | 11.90 | 3 | 15.08 | 17.63 | 0.26 |
| F2DMJ5 | F2DMJ5_HORVD | Bifunctional nuclease | 36780 | 9.38 | 6 | 2.13 | 13.14 | 0.49 |
| Q5ITG2 | Q5ITG2_HORVD | Grain softness protein | 18356 | 4.51 | 5 | 21.95 | 12.88 | 0.24 |
| Q8LLA7 | Q8LLA7_HORVU | Subtilisin-chymotrypsin inhibitor CI2C | 7953 | 5.09 | 13 | 56.76 | 8.56 | 0.07 |
| Q9SES6 | Q9SES6_HORVU | Non specific lipid transfer protein | 12332 | 8.51 | 9 | 56.20 | 6.56 | 0.08 |
| F2EE76 | F2EE76_HORVD | Non specific lipid transfer protein | 11827 | 8.30 | 1 | 11.22 | 6.02 | 0.07 |
| Q5UNP2 | Q5UNP2_HORVD | Non specific lipid transfer protein | 12354 | 8.96 | 8 | 52.42 | 5.33 | 0.07 |
| F2CSK4 | F2CSK4_HORVD | Glucose-ribitol dehydrogenase | 37492 | 9.41 | 18 | 31.41 | 4.15 | 0.16 |
| F2CUE9 | F2CUE9_HORVD | Glyceraldehyde-3-phosphate dehydrogenase (GAPDH) | 36490 | 6.78 | 8 | 25.52 | 2.54 | 0.09 |
| F2ECH4 | F2ECH4_HORVD | Late embryogenesis abundant protein | 38651 | 6.53 | 17 | 31.22 | 2.23 | 0.09 |
| F2DHH7 | F2DHH7_HORVD | Superoxide dismutase Cu Zn | 15089 | 5.80 | 3 | 31.58 | 2.06 | 0.03 |
| F2EEH9 | F2EEH9_HORVD | Non specific lipid transfer protein | 11409 | 8.74 | 4 | 38.46 | 1.69 | 0.02 |
| F2D4L0 | F2D4L0_HORVD | Glutathione S-transferase 3 | 23557 | 5.61 | 5 | 21.03 | 1.44 | 0.03 |
| F2D4W6 | F2D4W6_HORVD | Malate dehydrogenase | 35578 | 5.50 | 6 | 21.32 | 1.06 | 0.04 |
| F2EG29 | F2EG29_HORVD | Late embryogenesis abundant protein B19.3 (LEA5) | 14595 | 5.22 | 7 | 57.14 | 0.96 | 0.01 |
| F2EH61 | F2EH61_HORVD | Polyubiquitin | 51155 | 7.75 | 8 | 18.38 | 0.91 | 0.05 |
| Q850M2 | Q850M2_HORVD | Phosphoglycerate kinase | 31301 | 4.86 | 7 | 20.47 | 0.86 | 0.03 |
| **Embryo Globulins** | |  |  |  |  |  |  |  |
| Q03678 | Q03678_HORVU | Embryo globulin | 72209 | 6.83 | 16 | 15.23 | 67.61 | 4.88 |
| M0XUU4 | M0XUU4_HORVD | Globulin-1 S isoform (S7 globulin forming) | 47951 | 7.93 | 9 | 15.90 | 54.59 | 2.62 |
| P00924 | ENO1_YEAST | Enolase 1 EC 4 2 1 11 | 46642 | 6.15 | 57 | 42.43 | 50.00 | 2.34 |
| F2D483 | F2D483_HORVD | 40S ribosomal protein S8 | 25248 | 10.90 | 6 | 19.54 | 24.50 | 0.62 |
| M0Y075 | M0Y075_HORVD | Bowman Birk type trypsin inhibitor | 19176 | 8.02 | 10 | 28.49 | 18.21 | 0.37 |
| Q43770 | Q43770_HORVU | Oleosin | 16020 | 9.59 | 6 | 26.58 | 15.30 | 0.25 |
| F2CRF2 | F2CRF2_HORVD | Oleosin | 16091 | 9.59 | 9 | 18.06 | 13.50 | 0.22 |
| M0WXP0 | M0WXP0_HORVD | Oleosin | 16204 | 9.59 | 3 | 7.55 | 12.75 | 0.21 |
| Q9M4C9 | Q9M4C9_HORVU | Subtilisin-chymotrypsin inhibitor bci-7 | 7913 | 5.09 | 5 | 19.78 | 8.11 | 0.06 |
| F2E598 | F2E598_HORVD | Ribosomal S19e protein | 17073 | 10.25 | 8 | 27.10 | 4.73 | 0.08 |
| F2E598 | F2E598_HORVD | Ribosomal S19e protein | 17073 | 10.25 | 10 | 5.24 | 4.73 | 0.08 |
| F2E455 | F2E455_HORVD | 23 kDa jasmonate-induced protein | 19219 | 6.40 | 4 | 15.62 | 4.50 | 0.09 |
| Q42848 | Q42848_HORVU | Non specific lipid transfer protein | 12321 | 9.54 | 4 | 4.92 | 4.26 | 0.05 |
| Q9M4C9 | Q9M4C9_HORVU | Subtilisin-chymotrypsin inhibitor bci-7 | 7913 | 5.09 | 2 | 3.57 | 3.40 | 0.96 |
| F2CV88 | F2CV88_HORVD | 40S ribosomal protein S26-3 | 15682 | 11.44 | 2 | 4.50 | 3.20 | 0.05 |
| M0VYA0 | M0VYA0_HORVD | Type 1 Non Specific Lipid Transfer Protein Precursor | 12321 | 9.54 | 6 | 11.48 | 2.88 | 0.04 |
| O49872 | O49872_HORVU | 40S ribosomal protein S28-like | 7481 | 11.85 | 1 | 9.65 | 2.70 | 0.02 |
| M0WK21 | M0WK21_HORVD | GTPase OBG-like | 57960 | 4.53 | 5 | 6.39 | 1.71 | 0.10 |
| **Endosperm Albumins** | |  |  |  |  |  |  |  |
| P00924 | ENO1_YEAST | Enolase 1 EC 4 2 1 11 | 46642 | 6.15 | 72 | 38.07 | 50.00 | 2.34 |
| O24000 | O24000_HORVU | Alpha amylase trypsin inhibitor CMd3 | 18458 | 7.68 | 3 | 9.94 | 27.91 | 0.53 |
| P11643 | IAAD_HORVU | Alpha amylase trypsin inhibitor CMd | 18513 | 6.07 | 10 | 28.07 | 11.95 | 0.23 |
| P01086 | IAAE_HORVU | Alpha amylase trypsin inhibitor CMe | 16124 | 7.42 | 8 | 18.24 | 11.03 | 0.18 |
| E7BB45 | E7BB45_HORVD | Barley trypsin inhibitor CMc | 11241 | 7.44 | 11 | 56.73 | 10.49 | 0.12 |
| E7BB45 | E7BB45_HORVD | Barley trypsin inhibitor CMc | 11241 | 7.44 | 11 | 56.73 | 10.49 | 0.12 |
| P32936 | IAAB_HORVU | Alpha amylase trypsin inhibitor CMb | 16514 | 5.67 | 16 | 39.60 | 9.33 | 0.16 |
| P13691 | IAA2_HORVU | Alpha amylase inhibitor BDAI 1 | 16417 | 5.19 | 8 | 47.37 | 8.90 | 0.15 |
| Q546U1 | Q546U1_HORVU | Dimeric alpha amylase inhibitor Bdai | 16417 | 5.19 | 8 | 47.37 | 8.90 | 0.15 |
| F2EE76 | F2EE76_HORVD | Non specific lipid transfer protein | 11827 | 8.30 | 6 | 33.64 | 6.42 | 0.08 |
| F2EEH7 | F2EEH7_HORVD | Trypsin alpha amylase inhibitor | 11751 | 8.30 | 5 | 33.64 | 6.42 | 0.08 |
| P01545 | THNA_HORVU | Alpha hordothionin | 13587 | 6.17 | 5 | 22.05 | 6.12 | 0.09 |
| P28041 | IAAA_HORVU | Alpha amylase trypsin inhibitor CMa | 15489 | 5.84 | 7 | 35.86 | 5.68 | 0.09 |
| O49861 | O49861_HORVS | Alpha amylase trypsin inhibitor BTI CMe2 1 | 16212 | 6.89 | 9 | 42.57 | 3.53 | 0.06 |
| P16968 | IAA1_HORVU | Alpha amylase inhibitor BMAI 1 | 15805 | 5.15 | 5 | 23.97 | 3.51 | 0.06 |
| Q5URW5 | Q5URW5_HORVD | Hordoindoline a | 16472 | 8.13 | 9 | 63.09 | 3.31 | 0.06 |
| M0VFV9 | M0VFV9_HORVD | Alpha amylase trypsin inhibitor CMc | 15724 | 4.94 | 8 | 35.62 | 2.59 | 0.04 |
| I6TRT5 | I6TRT5_HORVU | B3 hordein | 31798 | 7.85 | 3 | 6.45 | 2.32 | 0.07 |
| I6TRT5 | I6TRT5_HORVU | B3 hordein | 31798 | 7.85 | 3 | 17.56 | 2.32 | 0.07 |
| A9E4R4 | A9E4R4_HORVU | Grain softness protein | 14223 | 4.36 | 5 | 44.53 | 1.87 | 0.03 |
| F2EE63 | F2EE63_HORVD | Thionin | 14562 | 6.71 | 6 | 28.68 | 1.87 | 0.03 |
| M0Z0D3 | M0Z0D3_HORVD | Malate dehydrogenase | 33153 | 5.33 | 3 | 10.03 | 1.76 | 0.06 |
| F2D4W6 | F2D4W6_HORVD | Malate dehydrogenase | 35578 | 5.50 | 4 | 20.12 | 1.39 | 0.05 |
| F2CWF9 | F2CWF9_HORVD | 40S ribosomal protein S28-like | 7459 | 11.58 | 7 | 33.85 | 1.23 | 0.01 |
| **Endosperm Globulins** | |  |  |  |  |  |  |  |
| P00924 | ENO1_YEAST | Enolase 1 | 46787 | 6.15 | 92 | 51.03 | 50.00 | 2.34 |
| O24000 | O24000_HORVU | Alpha amylase inhibitor CMd3 | 18458 | 7.68 | 2 | 19.30 | 10.99 | 0.21 |
| E7BB45 | E7BB45_HORVD | Barley trypsin inhibitor CMc | 11241 | 7.44 | 6 | 56.73 | 3.23 | 0.04 |
| F2EE63 | F2EE63_HORVD | Thionin | 14562 | 6.71 | 4 | 28.68 | 2.48 | 0.04 |
| Q40054 | Q40054_HORVU | D hordein | 75062 | 7.77 | 4 | 3.54 | 1.09 | 0.08 |
| **Endosperm Prolamins** | |  |  |  |  |  |  |  |
| P06471 | HOR3_HORVU | B3 hordein | 30176 | 7.53 | 26 | 19.32 | 76.23 | 2.34 |
| Q2XQF1 | Q2XQF1_HORVD | B hordein | 30136 | 7.86 | 38 | 27.92 | 76.23 | 2.33 |
| P00924 | ENO1_YEAST | Enolase 1 EC 4 2 1 11 | 46642 | 6.15 | 58 | 39.22 | 50.00 | 2.34 |
| Q70IB4 | Q70IB4_HORVU | Gamma 2 hordein | 29014 | 8.08 | 7 | 32.16 | 8.95 | 0.26 |
| E7BB45 | E7BB45_HORVD | Barley trypsin inhibitor CMc | 11241 | 7.44 | 8 | 86.54 | 6.29 | 0.07 |
| I6QP72 | I6QP72_HORVD | B hordein | 33481 | 7.06 | 8 | 12.41 | 5.81 | 0.20 |
| I6SW34 | I6SW34_HORVD | D hordein | 79303 | 7.96 | 11 | 6.96 | 4.15 | 0.33 |
| O24000 | O24000_HORVU | alpha amylase inhibitor CMd3 | 18458 | 7.68 | 4 | 34.50 | 3.13 | 0.06 |
| **Maltose Binding Protein (MBP5)** | |  |  |  |  |  |  |  |
| P00924 | ENO1_YEAST | Enolase 1 (Saccharomyces cerevisiae) | 46787 | 6.15 | 78 | 42.56 | 50.00 | 2.34 |
| **Alchool dehydrogenase 1 (Yeast)** | |  |  |  |  |  |  |  |
| P00924 | ENO1_YEAST | Enolase 1 EC 4 2 1 11 | 46642 | 6.15 | 80 | 39.22 | 50.00 | 2.34 |
| P00330 | ADH1_YEAST | Alcohol dehydrogenase I EC 1 1 1 1 | 36668 | 6.27 | 34 | 29.11 | 16.71 | 0.62 |

**Figure S1.** **SDS-PAGE of embryo globulins size separated (and electroeluted) by Gelfree 8100 and related DTZ stain after zinc overlay**. About 100 µg of embryo globulins (group 3) were separated by the Gelfree8100 fractionation station using 10% PAGE cartridge kit (Expedeon Inc., U.S.A.). The electroeluted fractions were further separated by SDS-PAGE (4-12% NuPAGE) which was run in duplicate. A first set was stained by Silver staining (panel A) and another set was blotted on PDVF membrane which were further treated for the Zinc/DTZ staining (panel B). Selected DTZ positive bands (red arrows) were cut from the PDVF membrane and identified by MS/MS (see Table S4). Lane description: (1) Maltose Binding Protein (MBP5, NEB England), (2) Alcohol dehydrogenase 1 (ADH1, Sigma-Aldrich), showing a partial proteolysis; and lanes (3-14) represent the electroeluted globulins from embryo extracted from the cartridge at different time points (minutes from the start of the run), respectively at 52, 54, 57, 61, 64, 68, 73, 80, 90, 105, 125, and 160 minutes, (15) HiMark Pre-stained High Molecular Weight Protein Standard (Invitrogen) showing the apparent MW in kDa.


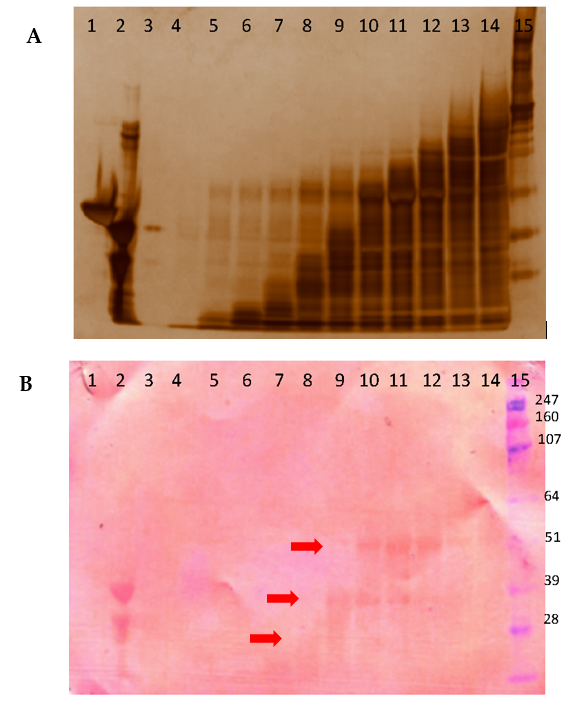


**Table S4** – **Total protein composition of a selected electroeluted protein fraction from embryo globulins**. All 12 electroeluted fractions have been analyzed by MS/MS and proteins have been identified using PEAKS software ver 6.0 (Bioinformatics Inc., Canada). Their abundance is given by the -10lgP parameter synonymous of the spectral abundance of the related peptides. The fraction 9 was chosen as the most representative since it contains three selected intense bands from supplementary figure S1. Those bands have been identified as follow: top band, embryo globulin (Q03678_HORVU); middle and lowest band, embryo globulin, Globulin-1S (M0XUU4_HORVD), Cupin 1 (F2EBM4_HORVD) Cupin 2 (F2CYL7_HORVD). The meaning of those apparent repetitions could rely on the fact that the intact globulins undergo proteolytic processing as for the wheat Glo-3 (Koziol et al., 2012).

| **Protein Group** | **Protein ID** | **Accession** | **-10lgP** | **Coverage (%)** | **#Peptides** | **#Unique** | **Mass** | **Description** |
| --- | --- | --- | --- | --- | --- | --- | --- | --- |
| **total 192 proteins** | | | | | | | | |
| **1** | 21373 | F2D5B2_HORVD | 248.09 | 55 | 18 | 4 | 59356.7 | ATP synthase subunit beta |
| **3** | 21374 | F2DJJ2_HORVD | 240.7 | 50 | 16 | 2 | 58916.2 | ATP synthase subunit beta |
| **6** | 21381 | F2D4A4_HORVD | 217.28 | 30 | 9 | 9 | 44177.2 | Ribosomal L4/L1E |
| **2** | 21375 | Q03678_HORVU | 213.32 | 31 | 17 | 17 | 72252.7 | Embryo globulin |
| **4** | 21376 | M0XUU4_HORVD | 205.16 | 41 | 10 | 8 | 47951.3 | Globulin-1 S |
| **7** | 21377 | A5CFY5_HORVD | 178.14 | 34 | 10 | 0 | 50045.3 | Beta tubulin 2 |
| **5** | 21378 | A5CFY9_HORVD | 173.75 | 34 | 10 | 2 | 50136.5 | Beta tubulin 6 |
| **5** | 21379 | A5CFY7_HORVD | 173.75 | 34 | 10 | 2 | 50308.7 | Beta tubulin 4 |
| **8** | 21391 | F2CRH2_HORVD | 171.27 | 23 | 6 | 6 | 49169.1 | Elongation factor 1-alpha |
| **8** | 21392 | Q6LAA4_HORVU | 171.27 | 23 | 6 | 6 | 49169.1 | Elongation factor 1-alpha |
| **10** | 21380 | A5CFY6_HORVD | 171.19 | 35 | 10 | 1 | 50238.6 | Beta tubulin 3 |
| **11** | 21382 | A5CFY4_HORVD | 166.93 | 31 | 9 | 0 | 50194.5 | Beta tubulin 1 |
| **12** | 21383 | A5CFY8_HORVD | 163.56 | 28 | 9 | 0 | 49971.3 | Beta tubulin 5 |
| **13** | 21394 | F2DIR3_HORVD | 154.41 | 26 | 7 | 7 | 29950 | Ribosomal_S4e_central |
| **14** | 21390 | A5CFZ0_HORVD | 152.12 | 26 | 7 | 0 | 49474.8 | Beta tubulin 7 |
| **16** | 21393 | F2EBM4_HORVD | 151.73 | 14 | 6 | 6 | 76923.6 | Cupin_1 |
| **9** | 21386 | F2CWX3_HORVD | 148.32 | 26 | 7 | 7 | 53520.9 | Cytochrome_P450 |
| **20** | 21396 | F2CR77_HORVD | 147.41 | 27 | 5 | 5 | 46943.8 | RNA-helicase_DEAD-box |
| **20** | 21397 | F2DPE3_HORVD | 147.41 | 27 | 5 | 5 | 46958.8 | DEAD box helicase family |
| **20** | 21398 | F2D2Z0_HORVD | 147.41 | 27 | 5 | 5 | 46944.8 | DEAD_ATP_HELICASE |
| **15** | 21401 | F2D483_HORVD | 143.75 | 28 | 5 | 5 | 25263.9 | 40S ribosomal protein S8 |
| **17** | 21411 | F2DSU6_HORVD | 139.48 | 18 | 5 | 5 | 36164.6 | G-protein_beta_WD-40 repeats |
| **36** | 21409 | F2D6I8_HORVD | 137.01 | 25 | 4 | 3 | 36611.9 | glyceraldehyde-3-phosphate dehydrogenase |
| **25** | 21406 | F2D448_HORVD | 135.85 | 28 | 4 | 4 | 30341.2 | Ribosomal_S5_D2-typ_fold |
| **27** | 21385 | F2CY68_HORVD | 134.47 | 26 | 8 | 8 | 58509.5 | peptidase M16 family (Zinc binding) |
| **24** | 21405 | F2D4W3_HORVD | 132.43 | 21 | 4 | 2 | 48231.8 | Enolase 1 |
| **32** | 21414 | F2CUE9_HORVD | 125.09 | 25 | 4 | 3 | 36513.7 | Glyceraldehyde 3-phosphate dehydrogenase GAPDH |
| **23** | 21402 | F2CYL7_HORVD | 124.34 | 14 | 5 | 5 | 55882.7 | Cupin 2 |
| **18** | 21403 | F2E8A9_HORVD | 120.93 | 21 | 5 | 5 | 40951.7 | PEROXIDASE_1 |
| **33** | 21417 | F2CR08_HORVD | 114.94 | 20 | 3 | 1 | 48058.6 | Enolase 2 |
| **41** | 21464 | F2DZZ0_HORVD | 114.18 | 12 | 2 | 2 | 46162.1 | DEHYDRIN |
| **10** | 2036 | A8WDM1_HORVD | 63.69 | 13 | 2 | 2 | 41006.6 | Dehydrin (Fragment) |
| **10** | 2012 | F2DZZ0_HORVD | 63.69 | 8 | 2 | 2 | 46162.14 | Predicted protein |
| **10** | 2037 | Q9SPA6_HORVU | 63.69 | 8 | 2 | 2 | 46156.14 | Dehydrin |
| **10** | 2038 | Q9ZTR5_HORVU | 63.69 | 11 | 2 | 2 | 47650.64 | Dehydrin 6 |
| **10** | 2039 | E7E2M3_HORVU | 63.69 | 11 | 2 | 2 | 47692.72 | Dehydrin 6 |
