# Supplementary material for: Enrichment and Identification of the Most Abundant Zinc Binding Proteins in Developing Barley Grains by Zinc-IMAC Capture and Nano LC-MS/MS
Source: Proteomes. 2018 Jan 17;6(1):3. doi: 10.3390/proteomes6010003 (PMC5874762; doi:10.3390/proteomes6010003)
Supplement: Supplementary file 1 [file proteomes-06-00003-s001.zip › proteomes-254233 supplementary 1.15/Supplementary files Article Dionisio final.pdf]

proteomes

Article

Enrichment and identification of the most abundant zinc binding proteins in developing barley grains by Zinc-IMAC capture and nano LC-MS/MS

Giuseppe Dionisio [1,*,†], Mohammad Nasir Uddin[2,†] and Eva Vincze [3,*]

SUPPLEMENTAL TABLES

TABLE OF CONTENTS:

Table S1: First round of MS/MS identification of captured zinc binding proteins for each microdissected fractions presented according to their four protein groups. …………………….. 2

Table S2: Second-round of MS/MS identification of captured zinc binding proteins for each microdissected fractions presented according to their four protein groups. ……………..……… 5

Table S3. Peptide mapping of the most abundant Zn-IMAC captured proteins (excel file)

Figure S1. SDS-PAGE of embryo globulins size separated (and electroeluted) by Gelfree 8100 and related DTZ stain after zinc overlay……………..……………………………………..……10

Table S4. Total protein composition of a selected electroeluted protein fraction from embryo globulins. ……………………………………………………………………………………..…… 11

Table S1. First round of MS/MS identification of captured zinc binding proteins for each microdissected fractions presented according to their four protein groups. The protein extraction has been performed without the use of DTT. The proteins have been ordered in a descending way based on their tryptic peptides spectral counting abundance.

Accession	ID	Description	MW (Da)	pI (pH)	Coverage (%)	Peptides
**Seed coats (group 1) albumins**						
P05698	RBL_HORVU	Ribulose bisphosphate carboxylase large chain	53044	6.22	20.04	12
M0VYA0	M0VYA0_HORVD	Type 1 Non Specific Lipid Transfer Protein Precursor	12321	9.54	25.41	5
P20145	NLTP2_HORVU	Probable non specific lipid transfer protein	10349	6.94	37.25	5
A1E9I8	ATPA_HORVU	ATP synthase subunit alpha chloroplastic	55260	6.32	14.48	5
F2CV78	F2CV78_HORVD	Non specific lipid transfer protein	9200	8.99	28.57	4
P01545	THNA_HORVU	Alpha hordothionin	13587	6.17	18.11	4
Q40004	RBS_HORVU	Ribulose bisphosphate carboxylase small chain chloroplastic	19407	8.94	27.01	3
**Embryo (group 3) albumins**						
M0ZDL8	M0ZDL8_HORVD	Late embryogenesis abundant protein	27486	5.56	31.16	18
F2EKY2	F2EKY2_HORVD	Late embryogenesis abundant protein	70517	7.34	32.95	14
M0Y075	M0Y075_HORVD	Bowman Birk type trypsin inhibitor	19176	8.02	42.46	14
Q5UNP2	Q5UNP2_HORVD	Non specific lipid transfer protein 2	12354	8.96	41.94	12
M0WCQ8	M0WCQ8_HORVD	Peptidyl-prolyl cis-trans isomerase	17354	8.72	32.92	11
F2CY84	F2CY84_HORVD	Non specific lipid transfer protein	12332	8.51	42.98	10
H2E688	H2E688_HORVU	Late embryogenesis abundant protein	9955	7.34	40.86	9
Q05190	LE19A_HORVU	Late embryogenesis abundant protein B19 1A	9955	7.34	59.14	8
P52572	REHY_HORVU	1 Cys peroxiredoxin PER1	23948	6.34	31.65	8
M0VYA0	M0VYA0_HORVD	Type 1 Non Specific Lipid Transfer Protein Precursor	12321	9.54	25.41	6
F2CY84	F2CY84_HORVD	Non specific lipid transfer protein	12332	8.51	33.06	5
M0UXX1	M0UXX1_HORVD	Acetyl-CoA acetyltransferase, cytosolic	38080	5.83	10.84	5
Q42848	Q42848_HORVU	Non specific lipid transfer protein	12321	9.54	25.41	4
A3RHQ9	A3RHQ9_HORVD	Glyceraldehyde 3 phosphate dehydrogenase	26273	6.62	10.61	3
P20145	NLTP2_HORVU	Non specific lipid transfer protein 2	10349	6.94	12.75	2
M0VN21	M0VN21_HORVD	Subtilisin-chymotrypsin inhibitor-2A	7961	5.06	20.27	2
Q9M4C9	Q9M4C9_HORVU	Subtilisin-chymotrypsin inhibitor-2A (bci 7), potato inhibitor like	7913	5.09	17.57	1
**Embryo (group 3) globulins**						
Q03678	Q03678_HORVU	Embryo globulin	72209	6.83	44.58	77
F2EKY2	F2EKY2_HORVD	Late Embryogenesis Abundant (LEA) protein	70517	7.34	40.20	51
F2DIX5	F2DIX5_HORVD	ATP-dependent DNA helicase 2 subunit ku80	79263	5.78	31.10	42
F2CT73	F2CT73_HORVD	Ribosomal protein L2_A	28173	11.49	50.19	33
F2CWX1	F2CWX1_HORVD	Elongation factor 2	85087	5.72	28.44	31
F2DIR3	F2DIR3_HORVD	Ribosomal protein S4e	29931	10.56	43.77	28
F2D483	F2D483_HORVD	40S ribosomal protein S8	25248	10.90	53.33	27

F2DIC8	F2DIC8_HORVD	RicinB_lectin_2	38713	6.29	54.18	26
F2EBM4	F2EBM4_HORVD	Globulin 2, Cupin, Vicilin-like (7S plant seed storage proteins)	76876	5.48	29.36	22
F2D4A4	F2D4A4_HORVD	60S ribosomal protein L4	44149	11.01	49.75	21
F2D5B2	F2D5B2_HORVD	ATP synthase subunit beta	59320	5.80	33.03	20
Q43769	Q43769_HORVU	Oleosin	18482	10.02	87.50	18
F2CVG5	F2CVG5_HORVD	mitochondrial import inner membrane translocase subunit Tim17/22/T23	18731	7.51	47.78	18
F2DSU6	F2DSU6_HORVD	Guanine nucleotide-binding protein beta subunit-like protein	36141	5.96	47.29	17
Q6LAA4	Q6LAA4_HORVU	Elongation factor 1 alpha	49137	9.48	24.83	16
F2D0U3	F2D0U3_HORVD	Ribosomal protein S19	17392	10.71	44.16	14
F2EEU3	F2EEU3_HORVD	Ribosomal protein S17_B	17804	11.00	35.22	13
F2D9E0	F2D9E0_HORVD	Ribosomal protein L10e	27580	11.55	34.43	12
F2D2Z0	F2D2Z0_HORVD	Eukaryotic initiation factor 4A-1	46914	5.15	32.61	12
Q43770	Q43770_HORVU	Oleosin	16020	9.59	29.11	11
F2EG94	F2EG94_HORVD	Ribosomal protein S7e	22146	10.13	48.96	11
F2D861	F2D861_HORVD	Ribosomal protein S12/S23	15650	10.91	42.25	10
F2CVF1	F2CVF1_HORVD	Ribosomal protein S15_S13e_A	17115	10.96	32.45	10
F2CV33	F2CV33_HORVD	Ribosomal protein L14	15005	10.93	39.29	10
F2DNP8	F2DNP8_HORVD	60S ribosomal protein L36	12522	11.86	34.23	9
F2CW48	F2CW48_HORVD	Ribosomal protein S25	12060	11.03	44.44	9
F2CSK4	F2CSK4_HORVD	Glucose and ribitol dehydrogenase-like protein	37492	9.41	27.38	9
F2CSC5	F2CSC5_HORVD	Ribosomal protein L19	24214	11.86	16.75	8
Q39999	Q39999_HORVU	Gamma thionin	8925	7.80	74.39	7
F2EJZ0	F2EJZ0_HORVD	Ribosomal protein L32e	15544	11.11	23.31	7
F2DT01	F2DT01_HORVD	Ribosomal protein L14	14701	11.08	45.32	7
F2DVU2	F2DVU2_HORVD	60S ribosomal protein L13	24114	11.35	24.52	7
F2D0I7	F2D0I7_HORVD	Ribosomal protein L2	24735	10.65	21.92	6
F2EFR1	F2EFR1_HORVD	40S ribosomal protein S6	28401	11.10	18.80	6
F2CUI5	F2CUI5_HORVD	Ribosomal protein L24e	18388	11.10	19.75	6
F2CUE9	F2CUE9_HORVD	Glyceraldehyde-3-phosphate dehydrogenase (GAPDH)	36490	6.78	28.19	6
F2EKP2	F2EKP2_HORVD	Ribosomal_S30	6908	12.43	17.74	5
Q9M4C9	Q9M4C9_HORVU	Subtilisin-chymotrypsin inhibitor bci-7 , potato inhibitor I like	7913	5.09	72.97	5
F2E8X4	F2E8X4_HORVD	Oleosin	16934	7.93	14.37	5
Q9ZTR5	Q9ZTR5_HORVU	Dehydrin 6	47622	8.66	18.13	5
F2EAU8	F2EAU8_HORVD	60S ribosomal protein L26-1	17532	11.54	24.20	5
F2CV88	F2CV88_HORVD	Ribosomal_S26e	15682	11.44	27.08	4
F2CZ65	F2CZ65_HORVD	Ribosomal protein L28e	16248	10.73	27.27	4
F2DB00	F2DB00_HORVD	Prohibitin-1, mitochondrial-like	30578	6.14	19.30	4
D0F044	D0F044_HORVU	Calmodulin Fragment	13235	3.98	31.90	3
F2CUP7	F2CUP7_HORVD	60S ribosomal protein L4-1	8908	10.34	32.50	3
F2DUY9	F2DUY9_HORVD	Ribosomal protein S19/S15	17166	10.50	26.80	2
**Endosperm (group 4) globulins**						

P01545	THNA_HORVU	Alpha hordothionin	13587	6.17	46.46	29
P21742	THNB_HORVU	Beta hordothionin	14592	6.71	35.29	20
I6SW34	I6SW34_HORVD	D hordein	79303	7.96	10.98	13
M0ULY1	M0ULY1_HORVD	Alpha amylase trypsin inhibitor CMb	15806	5.13	54.55	11
M0XH58	M0XH58_HORVD	Globulin-1 S2 isoform	46337	6.60	25.42	9
P28041	IAAA_HORVU	Alpha amylase trypsin inhibitor CMa	15489	5.84	33.10	7
M0Y046	M0Y046_HORVD	Gamma-thionin	8925	7.80	37.80	6
M0VYA0	M0VYA0_HORVD	Type 1 Non Specific Lipid Transfer Protein Precursor	12321	9.54	25.41	6
P16098	AMYB_HORVU	Beta amylase	59609	5.50	17.76	6
M0XDM7	M0XDM7_HORVD	RicinB lectin 2	26044	6.11	19.67	5
F2DQB4	F2DQB4_HORVD	Histone H2B	15095	10.55	18.84	4
P20145	NLTP2_HORVU	Non specific lipid transfer protein	10349	6.94	26.47	3
P06471	HOR3_HORVU	B3 hordein	30176	7.53	9.85	3
E7BB45	E7BB45_HORVD	Barley trypsin inhibitor CMc	11241	7.44	21.15	1
**Endosperm (group 4) hordeins**						
I6SW34	I6SW34_HORVD	D hordein	79303	7.96	11.51	43
P06471	HOR3_HORVU	B3 hordein	30176	7.53	9.85	22
I6QP72	I6QP72_HORVD	B hordein	33481	7.06	7.59	13
P01545	THNA_HORVU	Alpha hordothionin	13587	6.17	28.35	9
P17990	HOG1_HORVU	Gamma hordein 1	34714	7.95	11.80	8

Table S2. Second-round of MS/MS identification of captured zinc binding proteins for each microdissected fractions presented according to their four protein groups. The protein extraction has been performed with the use of DTT. The proteins have been ordered in a descending way based on their absolute quantification of their tryptic peptides.

Accession	ID	Description	MW (Da)	pI (pH)	Peptides	Coverage (%)	Amount (fmol)	Amount (ngrams)
**Seed Coats (group 1) Albumins**								
Q7YMR9	Q7YMR9_HORVS	Ribulose bisphosphate carboxylase large chain	53044	6.22	21	50.10	78.23	4.15
P00924	ENO1_YEAST	Enolase 1 EC 4 2 1 11	46642	6.15	73	37.39	50.00	2.34
F2EE76	F2EE76_HORVD	Non specific lipid transfer protein	11827	8.30	4	31.78	23.40	0.28
F2D6I8	F2D6I8_HORVD	Glyceraldehyde-3-phosphate dehydrogenase (GAPDH)	36588	6.75	13	34.72	21.30	0.78
F2DAU4	F2DAU4_HORVD	Translation elongation factor EFTu-EF1A	62011	7.40	15	32.14	13.10	0.81
F2EEH7	F2EEH7_HORVD	Trypsin alpha amylase inhibitor	11751	8.30	9	47.66	11.73	0.14
F2D714	F2D714_HORVD	Glyceraldehyde-3-phosphate dehydrogenase A, chloroplastic	42672	7.66	8	13.47	11.50	0.49
M0VFR1	M0VFR1_HORVD	LRR receptor-like serine/threonine-protein kinase RFK1	55251	7.85	4	10.02	10.01	0.56
F2DY47	F2DY47_HORVD	DNA directed RNA polymerase	133636	7.94	5	1.68	5.96	0.81
P11643	IAAD_HORVU	Alpha amylase trypsin inhibitor CMd	18513	6.07	4	37.43	5.87	0.11
P32936	IAAB_HORVU	Alpha amylase trypsin inhibitor CMb	16514	5.67	7	28.86	3.63	0.06
M0Z0D3	M0Z0D3_HORVD	Malate dehydrogenase	33153	5.33	3	14.89	3.13	0.10
P01086	IAAE_HORVU	Alpha amylase trypsin inhibitor CMe	16124	7.42	3	17.57	2.92	0.05
M0VDC1	M0VDC1_HORVD	SF-assemblin/beta giardin homologue	66115	10.19	5	6.81	2.49	0.17
M0VFV9	M0VFV9_HORVD	Alpha amylase trypsin inhibitor CMc	15724	4.94	3	23.29	1.85	0.03
**Seed Coats (group 1) Globulins**								
P00924	ENO1_YEAST	Enolase 1 EC 4 2 1 11	46642	6.15	64	40.83	50.00	2.34
F2CUP3	F2CUP3_HORVD	Histone H4	11402	11.88	6	29.13	6.35	0.07
M0VYA0	M0VYA0_HORVD	Type 1 Non Specific Lipid Transfer Protein Precursor	12321	9.54	13	41.80	5.47	0.07
P32936	IAAB_HORVU	Alpha amylase trypsin inhibitor CMb	16514	5.67	6	28.86	1.25	0.02
**Aleurone/Subaleurone (group 2) Albumins**								
P00924	ENO1_YEAST	Enolase 1 EC 4 2 1 11	46642	6.15	51	42.20	50.00	2.34
P07597	NLTP1_HORVU	Non specific lipid transfer protein 1	12293	8.31	17	54.70	41.18	0.53
F2EEH7	F2EEH7_HORVD	Trypsin alpha amylase inhibitor	11751	8.30	8	62.62	18.81	0.23
P01086	IAAE_HORVU	Alpha amylase trypsin inhibitor CMe	16124	7.42	11	18.24	15.57	0.26
P20145	NLTP2_HORVU	Non specific lipid transfer protein	10349	6.94	5	23.53	15.34	0.17
P11643	IAAD_HORVU	Alpha amylase trypsin inhibitor CMd	18513	6.07	7	53.22	14.06	0.27
P13691	IAA2_HORVU	Alpha amylase inhibitor BDAI 1	16417	5.19	6	37.50	10.76	0.18
P32936	IAAB_HORVU	Alpha amylase trypsin inhibitor CMb	16514	5.67	8	39.60	7.69	0.13
M0ZDP1	M0ZDP1_HORVD	Bifunctional nuclease	23979	8.40	6	7.76	7.30	0.18

P16968	IAA1_HORVU	Alpha amylase inhibitor BMAI 1	15805	5.15	4	26.71	3.81	0.06	
P28041	IAAA_HORVU	Alpha amylase trypsin inhibitor CMa	15489	5.84	4	44.83	2.44	0.04	
Q9FSI9	HINB1_HORVU	Hordoindoline B1	16110	8.30	9	23.13	2.38	0.04	
**Aleurone/Subaleurone (group 2) Globulins**									
P00924	ENO1_YEAST	Enolase 1 EC 4 2 1 11	46642	6.15	57	41.06	50.00	2.34	
P11643	IAAD_HORVU	Alpha amylase trypsin inhibitor CMd	18513	6.07	18	58.48	42.63	0.82	
P01545	THNA_HORVU	Alpha hordothionin	13587	6.17	17	39.37	38.52	0.56	
M0WPU2	M0WPU2_HORVD	Chromosome segregation ATPases	231298	4.83	27	11.10	30.23	7.03	
P32936	IAAB_HORVU	Alpha amylase trypsin inhibitor CMb	16514	5.67	12	39.60	27.84	0.48	
P13691	IAA2_HORVU	Alpha amylase inhibitor BDAI 1	16417	5.19	11	47.37	24.41	0.42	
P01086	IAAE_HORVU	Alpha amylase trypsin inhibitor CMe	16124	7.42	13	23.65	15.90	0.27	
P21742	THNB_HORVU	Beta hordothionin	14592	6.71	15	31.62	13.73	0.21	
E7BB45	E7BB45_HORVD	Barley trypsin inhibitor CMc	11241	7.44	7	56.73	10.23	0.12	
P28041	IAAA_HORVU	Alpha amylase trypsin inhibitor CMa	15489	5.84	7	35.86	8.32	0.13	
M0VYA0	M0VYA0_HORVD	Type 1 Non Specific Lipid Transfer Protein Precursor	12321	9.54	10	46.72	4.83	0.06	
P34951	IAAC_HORVU	Alpha amylase trypsin inhibitor CMc	15168	6.78	8	41.26	3.83	0.06	
I6SW34	I6SW34_HORVD	D hordein	79303	7.96	3	5.35	3.58	0.29	
Q9FSI9	HINB1_HORVU	Hordoindoline B1	16110	8.30	6	24.49	2.78	0.05	
P16968	IAA1_HORVU	Alpha amylase inhibitor BMAI 1	15805	5.15	4	26.71	2.69	0.04	
P07596	IAAS_HORVU	Alpha amylase subtilisin inhibitor	22149	7.88	11	46.31	2.09	0.05	
M0WYN5	M0WYN5_HORVD	Transposase (WRKY-GCM1 zinc finger)	227943	4.47	17	6.28	0.62	0.14	
**Aleurone/Subaleurone (group 2) Hordeins**									
I6TEV5	I6TEV5_HORVU	B3 hordein	35440	7.51	24	21.29	102.17	3.67	
P00924	ENO1_YEAST	Enolase 1	46787	6.15	75	47.83	50.00	2.34	
I6SJ17	I6SJ17_HORVD	Gamma 1 hordein	34714	7.95	18	23.61	38.08	1.34	
E7BB45	E7BB45_HORVD	Barley trypsin inhibitor CMc	11241	7.44	15	75.96	29.14	0.34	
Q3YAF9	Q3YAF9_HORVD	B hordein	34442	8.68	4	14.00	16.84	0.59	
Q5URW5	Q5URW5_HORVD	Hordoindoline a	16472	8.13	10	32.21	13.54	0.23	
I6TEV2	I6TEV2_HORVU	Gamma 3 hordein	33763	6.70	3	5.42	12.14	0.42	
F2EE63	F2EE63_HORVD	Thionin	14562	6.71	13	50.00	11.63	0.18	
G8FGD4	G8FGD4_HORVU	Limit dextrinase inhibitor	15962	6.93	10	51.70	11.63	0.17	
F2EA67	F2EA67_HORVD	D-Hordein	79273	7.78	11	10.98	10.60	0.92	
O24000	O24000_HORVU	Alpha amylase subtilisin inhibitor CMd3	18458	7.68	8	52.05	6.77	0.13	
F2EI31	F2EI31_HORVD	Dimeric alpha-amylase inhibitor (Bdai-1)	16605	5.19	9	71.43	6.67	0.12	
F2E235	F2E235_HORVD	Embryogenesis (PGG domain) transmembrane protein-like	48338	6.65	10	27.15	3.63	0.18	
F2DHS6	F2DHS6_HORVD	Serine/threonine CCR4-like cytokinin-regulated kinase	91450	7.82	9	17.10	1.75	0.16	
Q84VT9	Q84VT9_HORVU	Trypsin inhibitor	16076	6.80	3	8.84	1.53	0.03	
F2EFF9	F2EFF9_HORVD	FIZZY-RELATED 3-like cell cycle switch protein	52128	9.75	8	17.28	0.45	0.02	

Q8S409	Q8S409_HORVD	Hordoindoline a	15648	7.95	9	33.80	0.43	0.01
G1UH41	G1UH41_HORVS	Hordoindoline b1	16110	8.30	10	30.61	0.42	0.01
**Embryo Albumins**									
P00924	ENO1_YEAST	Enolase 1	46787	6.15	58	37.07	50.00	2.34
Q9M4C9	Q9M4C9_HORVU	Subtilisin-chymotrypsin inhibitor bci-7	7913	5.09	12	60.81	33.10	0.26
C9W327	C9W327_HORVD	Thionin	14611	7.21	9	36.50	26.99	0.42
B8K2C3	B8K2C3_HORVD	Transcription factor AP2 type (APETALA2 or EREBP)	14865	11.90	3	15.08	17.63	0.26
F2DMJ5	F2DMJ5_HORVD	Bifunctional nuclease	36780	9.38	6	2.13	13.14	0.49
Q5ITG2	Q5ITG2_HORVD	Grain softness protein	18356	4.51	5	21.95	12.88	0.24
Q8LLA7	Q8LLA7_HORVU	Subtilisin-chymotrypsin inhibitor CI2C	7953	5.09	13	56.76	8.56	0.07
Q9SES6	Q9SES6_HORVU	Non specific lipid transfer protein	12332	8.51	9	56.20	6.56	0.08
F2EE76	F2EE76_HORVD	Non specific lipid transfer protein	11827	8.30	1	11.22	6.02	0.07
Q5UNP2	Q5UNP2_HORVD	Non specific lipid transfer protein	12354	8.96	8	52.42	5.33	0.07
F2CSK4	F2CSK4_HORVD	Glucose-ribitol dehydrogenase	37492	9.41	18	31.41	4.15	0.16
F2CUE9	F2CUE9_HORVD	Glyceraldehyde-3-phosphate dehydrogenase (GAPDH)	36490	6.78	8	25.52	2.54	0.09
F2ECH4	F2ECH4_HORVD	Late embryogenesis abundant protein	38651	6.53	17	31.22	2.23	0.09
F2DHH7	F2DHH7_HORVD	Superoxide dismutase Cu Zn	15089	5.80	3	31.58	2.06	0.03
F2EEH9	F2EEH9_HORVD	Non specific lipid transfer protein	11409	8.74	4	38.46	1.69	0.02
F2D4L0	F2D4L0_HORVD	Glutathione S-transferase 3	23557	5.61	5	21.03	1.44	0.03
F2D4W6	F2D4W6_HORVD	Malate dehydrogenase	35578	5.50	6	21.32	1.06	0.04
F2EG29	F2EG29_HORVD	Late embryogenesis abundant protein B19.3 (LEA5)	14595	5.22	7	57.14	0.96	0.01
F2EH61	F2EH61_HORVD	Polyubiquitin	51155	7.75	8	18.38	0.91	0.05
Q850M2	Q850M2_HORVD	Phosphoglycerate kinase	31301	4.86	7	20.47	0.86	0.03
**Embryo Globulins**									
Q03678	Q03678_HORVU	Embryo globulin	72209	6.83	16	15.23	67.61	4.88
M0XUU4	M0XUU4_HORVD	Globulin-1 S isoform (S7 globulin forming)	47951	7.93	9	15.90	54.59	2.62
P00924	ENO1_YEAST	Enolase 1 EC 4 2 1 11	46642	6.15	57	42.43	50.00	2.34
F2D483	F2D483_HORVD	40S ribosomal protein S8	25248	10.90	6	19.54	24.50	0.62
M0Y075	M0Y075_HORVD	Bowman Birk type trypsin inhibitor	19176	8.02	10	28.49	18.21	0.37
Q43770	Q43770_HORVU	Oleosin	16020	9.59	6	26.58	15.30	0.25
F2CRF2	F2CRF2_HORVD	Oleosin	16091	9.59	9	18.06	13.50	0.22
M0WXP0	M0WXP0_HORVD	Oleosin	16204	9.59	3	7.55	12.75	0.21
Q9M4C9	Q9M4C9_HORVU	Subtilisin-chymotrypsin inhibitor bci-7	7913	5.09	5	19.78	8.11	0.06
F2E598	F2E598_HORVD	Ribosomal S19e protein	17073	10.25	8	27.10	4.73	0.08
F2E598	F2E598_HORVD	Ribosomal S19e protein	17073	10.25	10	5.24	4.73	0.08
F2E455	F2E455_HORVD	23 kDa jasmonate-induced protein	19219	6.40	4	15.62	4.50	0.09
Q42848	Q42848_HORVU	Non specific lipid transfer protein	12321	9.54	4	4.92	4.26	0.05
Q9M4C9	Q9M4C9_HORVU	Subtilisin-chymotrypsin inhibitor bci-7	7913	5.09	2	3.57	3.40	0.96
F2CV88	F2CV88_HORVD	40S ribosomal protein S26-3	15682	11.44	2	4.50	3.20	0.05
M0VYA0	M0VYA0_HORVD	Type 1 Non Specific Lipid Transfer Protein Precursor	12321	9.54	6	11.48	2.88	0.04

O49872	O49872_HORVU	40S ribosomal protein S28-like	7481	11.85	1	9.65	2.70	0.02
M0WK21	M0WK21_HORVD	GTPase OBG-like	57960	4.53	5	6.39	1.71	0.10
**Endosperm Albumins**								
P00924	ENO1_YEAST	Enolase 1 EC 4 2 1 11	46642	6.15	72	38.07	50.00	2.34
O24000	O24000_HORVU	Alpha amylase trypsin inhibitor CMd3	18458	7.68	3	9.94	27.91	0.53
P11643	IAAD_HORVU	Alpha amylase trypsin inhibitor CMd	18513	6.07	10	28.07	11.95	0.23
P01086	IAAE_HORVU	Alpha amylase trypsin inhibitor CMe	16124	7.42	8	18.24	11.03	0.18
E7BB45	E7BB45_HORVD	Barley trypsin inhibitor CMc	11241	7.44	11	56.73	10.49	0.12
E7BB45	E7BB45_HORVD	Barley trypsin inhibitor CMc	11241	7.44	11	56.73	10.49	0.12
P32936	IAAB_HORVU	Alpha amylase trypsin inhibitor CMb	16514	5.67	16	39.60	9.33	0.16
P13691	IAA2_HORVU	Alpha amylase inhibitor BDAI 1	16417	5.19	8	47.37	8.90	0.15
Q546U1	Q546U1_HORVU	Dimeric alpha amylase inhibitor Bdai	16417	5.19	8	47.37	8.90	0.15
F2EE76	F2EE76_HORVD	Non specific lipid transfer protein	11827	8.30	6	33.64	6.42	0.08
F2EEH7	F2EEH7_HORVD	Trypsin alpha amylase inhibitor	11751	8.30	5	33.64	6.42	0.08
P01545	THNA_HORVU	Alpha hordothionin	13587	6.17	5	22.05	6.12	0.09
P28041	IAAA_HORVU	Alpha amylase trypsin inhibitor CMa	15489	5.84	7	35.86	5.68	0.09
O49861	O49861_HORVS	Alpha amylase trypsin inhibitor BTI CMe2 1	16212	6.89	9	42.57	3.53	0.06
P16968	IAA1_HORVU	Alpha amylase inhibitor BMAI 1	15805	5.15	5	23.97	3.51	0.06
Q5URW5	Q5URW5_HORVD	Hordoindoline a	16472	8.13	9	63.09	3.31	0.06
M0VFV9	M0VFV9_HORVD	Alpha amylase trypsin inhibitor CMc	15724	4.94	8	35.62	2.59	0.04
I6TRT5	I6TRT5_HORVU	B3 hordein	31798	7.85	3	6.45	2.32	0.07
I6TRT5	I6TRT5_HORVU	B3 hordein	31798	7.85	3	17.56	2.32	0.07
A9E4R4	A9E4R4_HORVU	Grain softness protein	14223	4.36	5	44.53	1.87	0.03
F2EE63	F2EE63_HORVD	Thionin	14562	6.71	6	28.68	1.87	0.03
M0Z0D3	M0Z0D3_HORVD	Malate dehydrogenase	33153	5.33	3	10.03	1.76	0.06
F2D4W6	F2D4W6_HORVD	Malate dehydrogenase	35578	5.50	4	20.12	1.39	0.05
F2CWF9	F2CWF9_HORVD	40S ribosomal protein S28-like	7459	11.58	7	33.85	1.23	0.01
**Endosperm Globulins**								
P00924	ENO1_YEAST	Enolase 1	46787	6.15	92	51.03	50.00	2.34
O24000	O24000_HORVU	Alpha amylase inhibitor CMd3	18458	7.68	2	19.30	10.99	0.21
E7BB45	E7BB45_HORVD	Barley trypsin inhibitor CMc	11241	7.44	6	56.73	3.23	0.04
F2EE63	F2EE63_HORVD	Thionin	14562	6.71	4	28.68	2.48	0.04
Q40054	Q40054_HORVU	D hordein	75062	7.77	4	3.54	1.09	0.08
**Endosperm Prolamins**								
P06471	HOR3_HORVU	B3 hordein	30176	7.53	26	19.32	76.23	2.34
Q2XQF1	Q2XQF1_HORVD	B hordein	30136	7.86	38	27.92	76.23	2.33
P00924	ENO1_YEAST	Enolase 1 EC 4 2 1 11	46642	6.15	58	39.22	50.00	2.34
Q70IB4	Q70IB4_HORVU	Gamma 2 hordein	29014	8.08	7	32.16	8.95	0.26
E7BB45	E7BB45_HORVD	Barley trypsin inhibitor CMc	11241	7.44	8	86.54	6.29	0.07
I6QP72	I6QP72_HORVD	B hordein	33481	7.06	8	12.41	5.81	0.20

I6SW34	I6SW34_HORVD	D hordein	79303	7.96	11	6.96	4.15	0.33
O24000	O24000_HORVU	alpha amylase inhibitor CMd3	18458	7.68	4	34.50	3.13	0.06
**Maltose Binding Protein (MBP5)**								
P00924	ENO1_YEAST	Enolase 1 (Saccharomyces cerevisiae)	46787	6.15	78	42.56	50.00	2.34
**Alchool dehydrogenase 1 (Yeast)**								
P00924	ENO1_YEAST	Enolase 1 EC 4 2 1 11	46642	6.15	80	39.22	50.00	2.34
P00330	ADH1_YEAST	Alcohol dehydrogenase I EC 1 1 1 1	36668	6.27	34	29.11	16.71	0.62

Figure S1. SDS-PAGE of embryo globulins size separated (and electroeluted) by Gelfree 8100 and related DTZ stain after zinc overlay. About 100 µg of embryo globulins (group 3) were separated by the Gelfree8100 fractionation station using 10% PAGE cartridge kit (Expedeon Inc., U.S.A.). The electroeluted fractions were further separated by SDS-PAGE (4-12% NuPAGE) which was run in duplicate. A first set was stained by Silver staining (panel A) and another set was blotted on PDVF membrane which were further treated for the Zinc/DTZ staining (panel B). Selected DTZ positive bands (red arrows) were cut from the PDVF membrane and identified by MS/MS (see Table S4). Lane description: (1) Maltose Binding Protein (MBP5, NEB England), (2) Alcohol dehydrogenase 1 (ADH1, Sigma-Aldrich), showing a partial proteolysis; and lanes (3-14) represent the electroeluted globulins from embryo extracted from the cartridge at different time points (minutes from the start of the run), respectively at 52, 54, 57, 61, 64, 68, 73, 80, 90, 105, 125, and 160 minutes, (15) HiMark Pre-stained High Molecular Weight Protein Standard (Invitrogen) showing the apparent MW in kDa.

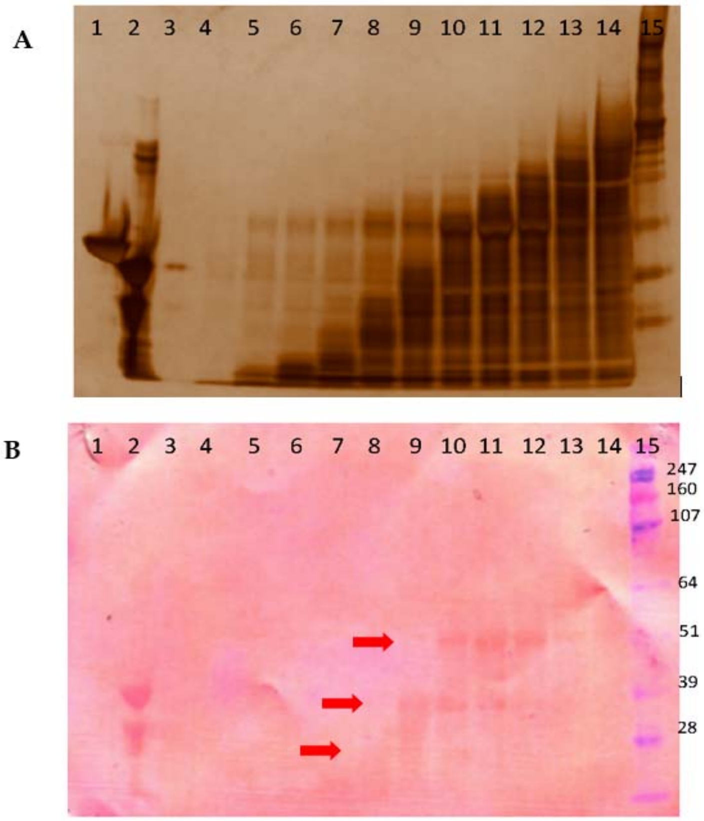

Table S4 – Total protein composition of a selected electroeluted protein fraction from embryo globulins. All 12 electroeluted fractions have been analyzed by MS/MS and proteins have been identified using PEAKS software ver 6.0 (Bioinformatics Inc., Canada). Their abundance is given by the -10lgP parameter synonymous of the spectral abundance of the related peptides. The fraction 9 was chosen as the most representative since it contains three selected intense bands from supplementary figure S1. Those bands have been identified as follow: top band, embryo globulin (Q03678_HORVU); middle and lowest band, embryo globulin, Globulin-1S (M0XUU4_HORVD), Cupin 1 (F2EBM4_HORVD) Cupin 2 (F2CYL7_HORVD). The meaning of those apparent repetitions could rely on the fact that the intact globulins undergo proteolytic processing as for the wheat Glo-3 (Koziol et al., 2012).

Protein Group	Protein ID	Accession	-10lgP	Coverage (%)	#Peptides	#Unique	Mass	Description
**total 192 proteins**								
**1**	21373	F2D5B2_HORVD	248.09	55	18	4	59356.7	ATP synthase subunit beta
**3**	21374	F2DJJ2_HORVD	240.7	50	16	2	58916.2	ATP synthase subunit beta
**6**	21381	F2D4A4_HORVD	217.28	30	9	9	44177.2	Ribosomal L4/L1E
**2**	21375	Q03678_HORVU	213.32	31	17	17	72252.7	Embryo globulin
**4**	21376	M0XUU4_HORVD	205.16	41	10	8	47951.3	Globulin-1 S
**7**	21377	A5CFY5_HORVD	178.14	34	10	0	50045.3	Beta tubulin 2
**5**	21378	A5CFY9_HORVD	173.75	34	10	2	50136.5	Beta tubulin 6
**5**	21379	A5CFY7_HORVD	173.75	34	10	2	50308.7	Beta tubulin 4
**8**	21391	F2CRH2_HORVD	171.27	23	6	6	49169.1	Elongation factor 1-alpha
**8**	21392	Q6LAA4_HORVU	171.27	23	6	6	49169.1	Elongation factor 1-alpha
**10**	21380	A5CFY6_HORVD	171.19	35	10	1	50238.6	Beta tubulin 3
**11**	21382	A5CFY4_HORVD	166.93	31	9	0	50194.5	Beta tubulin 1
**12**	21383	A5CFY8_HORVD	163.56	28	9	0	49971.3	Beta tubulin 5
**13**	21394	F2DIR3_HORVD	154.41	26	7	7	29950	Ribosomal_S4e_central
**14**	21390	A5CFZ0_HORVD	152.12	26	7	0	49474.8	Beta tubulin 7
**16**	21393	F2EBM4_HORVD	151.73	14	6	6	76923.6	Cupin_1
**9**	21386	F2CWX3_HORVD	148.32	26	7	7	53520.9	Cytochrome_P450
**20**	21396	F2CR77_HORVD	147.41	27	5	5	46943.8	RNA-helicase_DEAD-box
**20**	21397	F2DPE3_HORVD	147.41	27	5	5	46958.8	DEAD box helicase family
**20**	21398	F2D2Z0_HORVD	147.41	27	5	5	46944.8	DEAD_ATP_HELICASE
**15**	21401	F2D483_HORVD	143.75	28	5	5	25263.9	40S ribosomal protein S8
**17**	21411	F2DSU6_HORVD	139.48	18	5	5	36164.6	G-protein_beta_WD-40 repeats
**36**	21409	F2D6I8_HORVD	137.01	25	4	3	36611.9	glyceraldehyde-3-phosphate dehydrogenase
**25**	21406	F2D448_HORVD	135.85	28	4	4	30341.2	Ribosomal_S5_D2-typ_fold
**27**	21385	F2CY68_HORVD	134.47	26	8	8	58509.5	peptidase M16 family (Zinc binding)
**24**	21405	F2D4W3_HORVD	132.43	21	4	2	48231.8	Enolase 1

**32**	21414	F2CUE9_HORVD	125.09	25	4	3	36513.7	Glyceraldehyde 3-phosphate dehydrogenase GAPDH
**23**	21402	F2CYL7_HORVD	124.34	14	5	5	55882.7	Cupin 2
**18**	21403	F2E8A9_HORVD	120.93	21	5	5	40951.7	PEROXIDASE_1
**33**	21417	F2CR08_HORVD	114.94	20	3	1	48058.6	Enolase 2
**41**	21464	F2DZZ0_HORVD	114.18	12	2	2	46162.1	DEHYDRIN
**10**	2036	A8WDM1_HORVD	63.69	13	2	2	41006.6	Dehydrin (Fragment)
**10**	2012	F2DZZ0_HORVD	63.69	8	2	2	46162.14	Predicted protein
**10**	2037	Q9SPA6_HORVU	63.69	8	2	2	46156.14	Dehydrin
**10**	2038	Q9ZTR5_HORVU	63.69	11	2	2	47650.64	Dehydrin 6
**10**	2039	E7E2M3_HORVU	63.69	11	2	2	47692.72	Dehydrin 6
